# Towards making the fields talks: A real-time cloud enabled IoT crop management platform for smart agriculture

**DOI:** 10.3389/fpls.2022.1030168

**Published:** 2023-01-04

**Authors:** Navod Neranjan Thilakarathne, Muhammad Saifullah Abu Bakar, Pg Emerolylariffion Abas, Hayati Yassin

**Affiliations:** Faculty of Integrated Technologies, Universiti Brunei Darussalam, Brunei

**Keywords:** internet of things, IoT, agriculture, smart agriculture, precision agriculture, cloud, sensors

## Abstract

Agriculture is the primary and oldest industry in the world and has been transformed over the centuries from the prehistoric era to the technology-driven 21^st^ century, where people are always solving complex problems with the aid of technology. With the power of Information and Communication Technologies (ICTs), the world has become a global village, where every digital object that prevails in the world is connected to each other with the Internet of Things (IoT). The fast proliferation of IoT-based technology has revolutionized practically every sector, including agriculture, shifting the industry from statistical to quantitative techniques. Such profound transformations are reshaping traditional agricultural practices and generating new possibilities in the face of various challenges. With the opportunities created, farmers are now able to monitor the condition of crops in real time. With the automated IoT solutions, farmers can automate tasks in the farmland, as these solutions are capable of making precise decisions based on underlying challenges and executing actions to overcome such difficulties, alerting farmers in real-time, eventually leading to increased productivity and higher harvest. In this context, we present a cloud-enabled low-cost sensorized IoT platform for real-time monitoring and automating tasks dealing with a tomato plantation in an indoor environment, highlighting the necessity of smart agriculture. We anticipate that the findings of this study will serve as vital guides in developing and promoting smart agriculture solutions aimed at improving productivity and quality while also enabling the transition to a sustainable environment.

## 1 Introduction

At the beginning of the 21st century, we witnessed many technological revolutions, and all our lives have been changed to a greater extent with the absorption of these technologies in our daily tasks (Reddy et al., 2020). Owing to the contributions offered by these different technologies, many works that require a lot of human effort can now be carried out with less human intervention and supervision with greater flexibility and convenience (Reddy et al., 2020; Ahmed et al., 2022). Almost all domains, including healthcare, military, education, surveillance, and transportation, have absorbed these technologies and used them to offer convenient and flexible services to the people (Bates et al., 2021; Ahmed et al., 2022). Among these technologies, ICT plays a pivotal role in interconnecting digital devices as well as connecting these devices to the Internet (Reddy et al., 2020; Forcén-Muñoz et al., 2021; Ahmed et al., 2022; Smart agriculture, 2022). The exponential growth of ICT technologies has paved the way for a more advanced set of technologies, including the IoT, Cloud Computing, Fog Computing, Edge Computing, Artificial Intelligence (AI), Mobile Computing, Software Defined Networking (SDN), and fifth-generation broadband cellular networks (5G) (Bates et al., 2021; Forcén-Muñoz et al., 2021; Mohammed et al., 2021; Ahmed et al., 2022; GlobeNewswire, 2022; The digitization of the European Agricultural Sector, 2022; Theparod and Harnsoongnoen, 2022) which leverage almost all the technological infrastructure onto the next level, paving the way for a technological revolution of the century.

Among all these collations of fruitful technologies, the IoT is closer to our daily life by allowing ubiquitous connectivity of devices, connecting digital devices all around the world to the WWW through Machine to Machine (M2M) communication (Reddy et al., 2020; Ahmed et al., 2022). In general, IoT is a cutting-edge technology for monitoring and controlling devices from anywhere in the world (Forcén-Muñoz et al., 2021; Smart agriculture, 2022), and it is making a significant impact in different domains, including agriculture, transportation, military, smart cities, and healthcare domains, by facilitating automation of many processes (Ahmed et al., 2022), where the adoption of IoT has made man’s life easier and more pleasant.

As the world’s principal and primary industry, agriculture need to balance the food requirements of humankind as well as the production of essential raw materials for many industries (Walter et al., 2017; Theparod and Harnsoongnoen, 2022). It is the most important and basic vocation that many people all around the world are currently involved in (Reddy et al., 2020). Over the centuries, traditional agriculture has transformed in line with the social and cultural changes along with the technological revolutions, and as of now, many of the conventional agricultural methods are executed with the involvement of these technologies. Autonomous robotic vehicles have been developed for performing different agrarian tasks, including mechanical weeding, spraying fertilizer, and pesticides (Forcén-Muñoz et al., 2021; Mohammed et al., 2021; Ahmed et al., 2022; Smart agriculture, 2022). According to the latest reports offered by the World Bank and United Nations (Reddy et al., 2020), it is estimated that agricultural food production needs to increase by 50%-90% by 2050 to meet the future demand for food production and to feed the growing world population by mid-century (Smart agriculture, 2022). Undoubtedly, this is practically a challenging task owing to various challenges such as climate change and crop and pest diseases (Mohammed et al., 2021; Smart agriculture, 2022; The digitization of the European Agricultural Sector, 2022). However, the adoption of IoT and other related enabling technologies in smart agriculture provides a lot of avenues for overcoming such challenges posed by traditional agriculture. Collectively, the applications of IoT and other enabling technologies in agriculture are known as smart agriculture or interchangeably known as smart farming.

Smart agriculture offers the potential to automate many agricultural fieldworks with minimal human intervention requiring a minimum amount of input resources such as less amount of fertilizer, pesticides, and water supply as the resources can be managed effectively as opposed to traditional agriculture (Reddy et al., 2020). The backbone of smart agriculture constitutes mainly of IoT at its core but is supplemented by other related technologies, including cloud computing, AI, fog computing, edge computing, big data, SDN, and underlying communication technologies, such as 5G and Wi-Fi (Mohammed et al., 2021; Smart agriculture, 2022). Altogether these technologies constitute complex cyber-physical systems and digital twins for smart agricultural applications capable of superseding traditional agricultural methodologies (Reddy et al., 2020). These smart agriculture applications range from applications for overall farm management (Reddy et al., 2020), crop condition monitoring (Bates et al., 2021; Forcén-Muñoz et al., 2021; Smart agriculture, 2022), soil condition monitoring (Reddy et al., 2020), livestock monitoring (The digitization of the European Agricultural Sector, 2022; Mohammed et al.,2021; Theparod and Harnsoongnoen, 2022), pest and plant disease monitoring (Mohammed et al., 2021; GlobeNewswire, 2022; The digitization of the European Agricultural Sector, 2022; Theparod and Harnsoongnoen, 2022), fruit quality monitoring (Mohammed et al., 2021; GlobeNewswire, 2022; The digitization of the European Agricultural Sector, 2022), and bee colony management (Mohammed et al., 2021; GlobeNewswire, 2022). Overall, these applications enable farmers to remotely monitor, coordinate and control and make timely and precise decisions, maximizing food production while at the same time reducing food losses and expenses, which are essential in light of the rising global need for agricultural foods (GlobeNewswire, 2022; The digitization of the European Agricultural Sector, 2022). Hence, researchers and organizations have been working on creating innovative smart agricultural solutions that allow remote monitoring and controlling of farms to improve the productivity of global agricultural food production.

**Figure 1:**
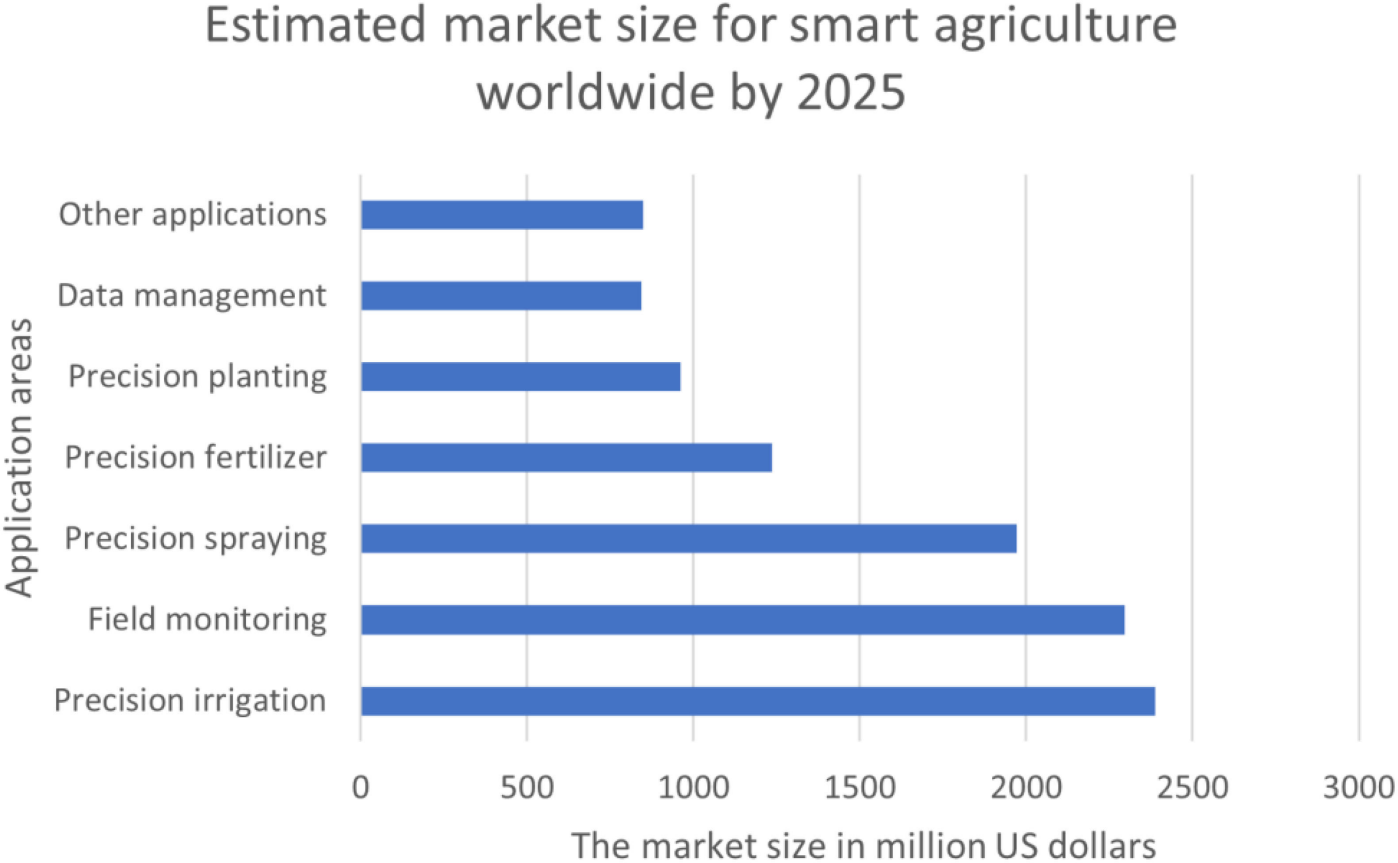
Estimated market size for smart agriculture by 2025 *(* The digitization of the European Agricultural Sector, 2022 *)*.

According to the latest statistics (GlobeNewswire, 2022; The digitization of the European Agricultural Sector, 2022), the global smart agriculture market is expected to reach 34.1 billion US dollars by 2026. The increasing world population, advancement of modern technologies, like IoT and AI, the popularity of large-scale farming, the acceptance of using modern technologies for livestock management, increased investments, and safety concerns caused by COVID-19 global pandemics are some of the key driving factors that help in boosting smart agricultural production. **Figure 1** showcases smart agriculture’s application-wise estimated market size by 2025 (The digitization of the European Agricultural Sector, 2022). The rise of automated farming in controlled environments, sustainable green agriculture, and expectations towards quality and higher harvest, which require fewer resources, are expected to contribute a lot towards adopting smart agricultural practices over traditional farming practices.

Despite the growth of the market, however, the adoption of smart agricultural solutions are currently still at a low rate, owing to a variety of reasons, including reluctance by some farmers to move with technologies and trend, higher initial investment, tendency to stick with traditional farming methods and reachability issues to reach out farmers in remote areas (Schwarz et al., 2014; Walter et al., 2017; Theparod and Harnsoongnoen, 2022). However, new research is always being carried out to overcome most of these issues (Schwarz et al., 2014; Walter et al., 2017; Mohammed et al., 2021).

As of now, many research studies in the field of smart agriculture have proposed different novel solutions and methodologies to address various research problems, including the prediction of daily climate for the next crop cycle and the amount of harvest in the next growth cycle (Chlingaryan et al., 2018; How to grow tomatoes indoors, 2022; Akhterand and Sofi,2021; Herman et al., 2019; Cordeiro et al., 2022). Various smart agriculture startup companies are established worldwide to reach out to more farmers and widen the market. As per the literature (Mehra et al., 2018; Herman et al., 2019; Junior et al., 2022), the key technical problems encountered in developing such solutions can be mainly apportioned into hardware, software, and networking and communication challenges. Hardware challenges include challenges related to the implementation of hardware, hardware capacity (e.g., size, memory, and performance), and challenges that may arise from the device’s operational environment, as often these devices need to be deployed under harsh environmental conditions (Bates et al., 2021; Ahmed et al., 2022). Software-level challenges may arise from software bugs, configuration issues, software vulnerabilities, and underlying software platform issues (Forcén-Muñoz et al., 2021; Smart agriculture, 2022). Lastly, networking and communication challenges include underlying networking infrastructure issues when reaching out to remote areas, communication protocol-level issues, and issues related to data transmission range (Mohammed et al., 2021; GlobeNewswire, 2022; The digitization of the European Agricultural Sector, 2022).

Taking all of these concerns into account, we aim to develop a novel real-time cloud-enabled low-cost IoT crop management platform for monitoring and automating indoor tomato plantations. This is done to promote a low-cost but reliable and convenient smart agricultural solution for indoor plantations. We relied on low-cost IoT sensors and open-source technologies for the platform’s design. Additionally, the developed platform should be convenient for farmers residing in urban areas who want to grow their own produce despite space restrictions. Even though previous researchers have used related technologies, they have concentrated on particular applications, their implementations, or technical elements, as opposed to our study. In contrast, in our work, we focus on configuring and deploying our platform with a real plantation to check the feasibility and reliability of the proposed platform. In this regard, the main contribution of the study is outlined below.

- Propose a cloud-enabled real-time monitoring platform for monitoring the environmental and soil condition of indoor tomato plantation along with automating irrigation and lighting conditions.
- Design and implement the platform with low-cost IoT sensors and actuators with open-source technologies.
- Provide our perspectives on how the platform can be extended towards indoor plant condition monitoring and urban farming where the resources are highly limited.
- Provide a comparative analysis of similar research work to differentiate our work from theirs.

The paper is organized in the following manner. Following the introduction, we highlight the necessity of smart agriculture along with enabling technologies. In the third section, the methodology of designing our platform is highlighted, followed by the results obtained after successful implementation with a brief discussion of the work. Section five compares similar research work related to smart agriculture to the proposed platform. The last section concludes the paper, highlighting future directions.

## 2 Why smart agriculture?

The agricultural sector has seen many revolutions over the years, beginning with the domestication of plants and animals thousands of years ago, progressing to the systematic use of machinery and instruments a few hundred years ago, and the use of man-made pesticides and fertilizers a few decades ago (Walter et al., 2017). At present, the agricultural sector is again undergoing another mega revolution triggered by the increased usage of ICT technologies, which started at the beginning of the 21st century. Autonomous agrarian equipment for farming tasks, including land preparation, sowing, weeding, fertilizer sprinkling, and fruit harvesting, have already been utilized to facilitate the agriculture processes (Reddy et al., 2020; Ahmed et al., 2022). Taken together, these technological advancements represent a technological revolution that will result in disruptive changes in the agricultural domain. This tendency applies to farming not just in developed nations but also in developing countries, where ICT deployments are accelerating and might become game changers in the future. This growth of ICT has fueled the flourishment of more advanced technologies, such as IoT. The rise of IoT, a key ICT breakthrough, has offered opportunities in enhancing practically every industry conceivable (Bates et al., 2021; Ahmed et al., 2022), not least the agricultural sector.

In agriculture, IoT is not only providing answers to frequently time-consuming and tiresome activities, but it has also completely transformed the way people think about agriculture. In this regard, this section is entirely devoted to highlighting the necessity of smart agriculture and offers a brief overview of enabling technologies of smart agriculture.

### 2.1 Smart agriculture cycle

According to references (Bates et al., 2021; Forcén-Muñoz et al., 2021; Mohammed et al., 2021; Ahmed et al., 2022; Smart agriculture, 2022; The digitization of the European Agricultural Sector, 2022; Theparod and Harnsoongnoen, 2022), smart agriculture refers to the management of farms via the utilization of an advanced set of technologies, including IoT, AI, cloud computing, and robotics, to improve quantity and quality of harvest whilst optimizing the utilization of resources, such as labor and raw materials. To optimize the agricultural processes, IoT devices are deployed on the farming land for continuous collection and analysis of environmental data, allowing farmers to respond rapidly and effectively to changes in ambient environments (Walter et al., 2017; Mohammed et al., 2021; The digitization of the European Agricultural Sector, 2022). In general, smart agriculture follows the following cycle (Walter et al., 2017; Mohammed et al., 2021; Theparod and Harnsoongnoen, 2022).

1. Observation – IoT sensors deployed in the field record data from crops, soil, atmosphere, and livestock.
2. Diagnostics –The sensor values collected from the observation phase are sent to remote data analytics servers or cloud-hosted platforms and compared with pre-determined business logic to ascertain the condition of the examined object and identify any deficiencies or needs.
3. Decisions – In the event of anomaly detection, the IoT platform’s user and/or AI-driven components determine whether location-specific treatment is necessary.
4. Action - After end-user evaluation and action, the cycle repeats itself.

A smart agriculture solution provides the agricultural sector with new levels of control and automated decision-making, allowing for a coherent ecosystem. With the current pace of development of smart agriculture, it is now feasible to construct a farm-wide sensor network (Chlingaryan et al., 2018; Herman et al., 2019; Akhter and Sofi, 2022; How to grow tomatoes indoors, 2022; Cordeiro et al., 2022) that allows for practically continuous round-the-clock monitoring of a farm. Consequently, theoretical and practical frameworks have been developed to link the state of crops, soil, and farm animals with production inputs, such as water, fertilizer, pesticides, and plant medications (Mehra et al., 2018; Kashyap et al., 2018; Herman et al., 2019; Parihar, 2019; Junior et al., 2022). Adoption of smart agriculture practices can make agriculture more profitable for farmers over traditional agriculture practices by optimizing most human efforts and input resources. Further, crop cultivation can be optimized using optimum, site-specific weather predictions (Bates et al., 2021; Forcén-Muñoz et al., 2021; Ahmed et al., 2022), yield estimates (Bates et al., 2021; Ahmed et al., 2022), and likelihood maps for illnesses and catastrophes based on meteorological and climate data (Badamasi, 2014; Arduino, 2015; Salim et al., 2015; Suryawinata et al., 2017; Kashyap et al., 2018). Site-specific smart information offers new insurance and economic possibilities for the whole value chain of agriculture, offering numerous benefits for farmers (Eller and Denoth, 1996; Cucus and Febrianti, 2017; Sunday et al., 2020; Saha et al., 2021). Naturally, collecting farming-related data via automated sensors reduces the time required for resource prioritization and administrative oversight (Latha et al., 2016; Banerjee et al., 2017; Gay, 2018; Abbas et al., 2020; Thinger.io, 2022). At the same time, smart agriculture also decreases the environmental impact of farming. Nonetheless, smart agriculture can potentially minimize site-specific use of inputs, including fertilizers and pesticides, which results in the reduction of greenhouse gas emissions, and this can also increase consumer acceptability (Max et al., 2009; Lee et al., 2014; Leyva et al., 2015; Shamshiri et al., 2018; Luis Bustamante et al., 2019).

Urbanization is inevitable as the world’s population continues to expand, leading to the loss of arable land and reduced water supplies. Limitations of these resources may lead to problems with food production in metropolitan areas (Mohammed et al., 2021). Thus, urban farming, which necessitates the use of smart agricultural solutions for the optimum utilization of scarce resources, has emerged as a unique solution to the challenges of land and water shortages caused by urbanization (Banerjee et al., 2017; Gay, 2018; Sunday et al., 2020; Abbas et al., 2020; Saha et al., 2021). Smart agricultural solutions focus on real-time monitoring and automation of necessary works, saving time, space, and cost, and offering higher conveniences to the stakeholders involved in urban farming.

IoT constitutes the core of a smart agriculture solution with collections of sensors, actuators, agriculture robots, and interconnected devices. Other enabling technologies that help strengthen and provide a stronger foundation for smart agriculture include big data from the collection of a vast amount of sensor data, AI for inferencing the meaning of the collected agricultural farming data, cloud computing for convenient and on-demand computing resources, mobile computing for allowing farmers to connect anytime with the smart agricultural solutions, and underlying communication technologies for data communication (Lee et al., 2014; Doshi et al., 2019; Ratnaparkhi et al., 2020). Even though these technologies contribute significantly to the creation of smart agricultural services and applications, without the participation of IoT, none of the services and applications would be useable since IoT is required for data collection. As such, it is evident that IoT plays an important role as the foundation of smart agriculture.

Thus, in the next sub-section, we plan to go further on the role of IoT in smart agriculture since neither the other supporting technologies in smart agriculture nor smart agriculture would exist without IoT.

### 2.2 IoT in smart agriculture

The IoT is known to be the present and future of everything and will continue to affect the lives of everyone in the world by bringing intelligence to everything (Bates et al., 2021). Its primary objective is to accomplish the creation of a vast network via the interconnection of a wide variety of sensing devices (Forcén-Muñoz et al., 2021). In simplest terms, IoT is a self-configuring network comprised of various inter-connected devices. Smart agriculture with IoT transforms traditional farming practices by making them more efficient and cost-effective for farmers while also lowering crop loss (Bates et al., 2021; Forcén-Muñoz et al., 2021). At present, most agricultural operations are often hindered by a variety of reasons, including global pandemics such as COVID-19, climate changes, lack of trained personnel, lack of faith in technology, and expensive capital costs (Mekala and Viswanathan, 2017; Sushanth and Sujatha, 2018; Ayaz et al., 2019; Thilakarathne et al., 2021; Growing tomatoes from seed, 2022), where IoT is recognized as a record-breaking technology that can offer solutions to most of such challenges.

In smart agriculture, IoT sensors deployed in the farming field and livestock first collect data from the environment in which they are deployed, and then the gathered data are sent to the cloud or data analytics servers through wireless and wired communication media (Schwarz et al., 2014; Walter et al., 2017), in accordance with the cycle of smart agriculture as explained in the previous subsection.

**Figure 2:**
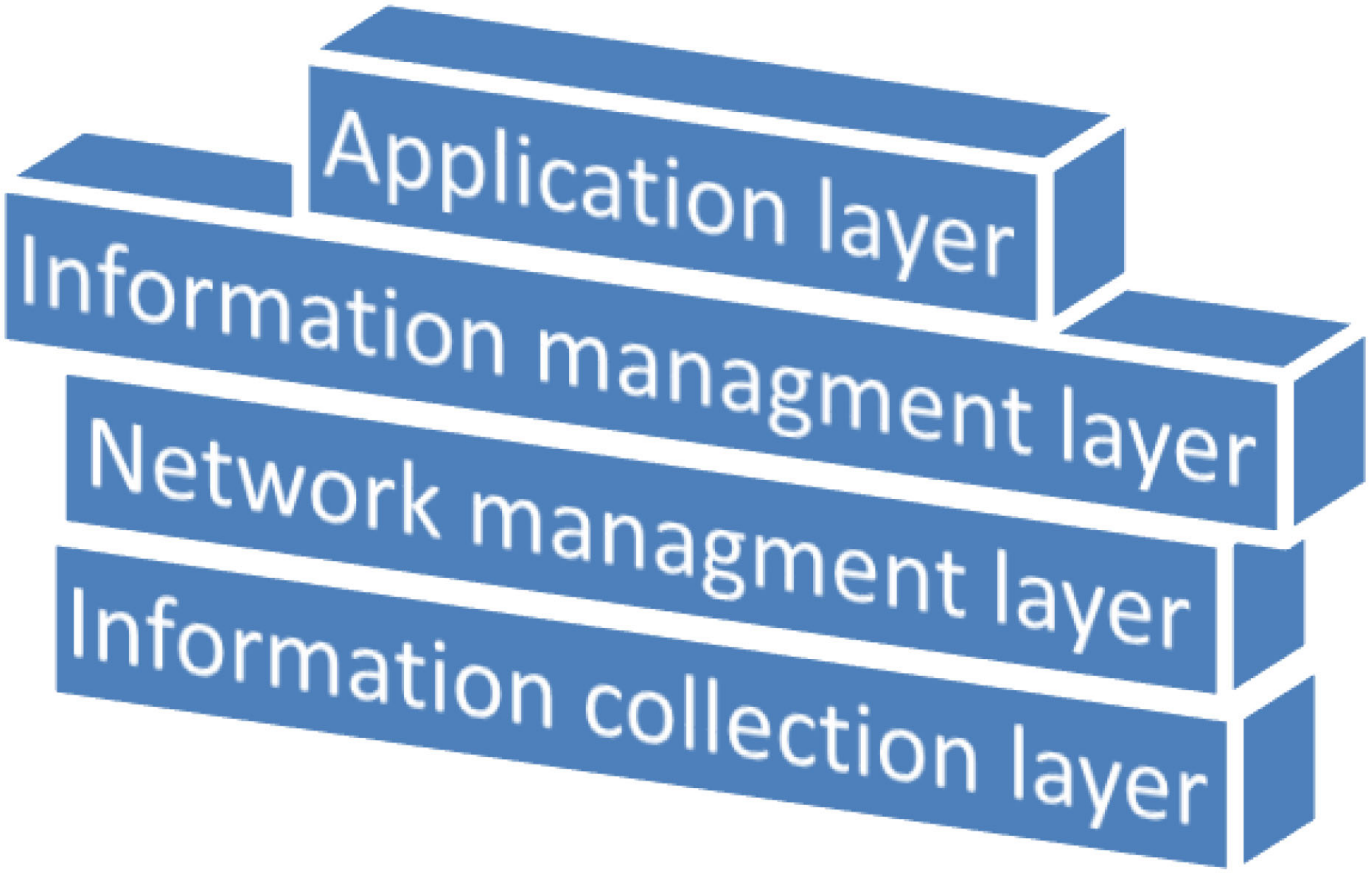
Layers of the IoT architecture in smart agriculture.

IoT is made up of several physical devices in the smart agricultural domain and has a four-layer architecture (Schwarz et al., 2014; Walter et al., 2017; Bates et al., 2021), as shown in **Figure 2**. The first layer is the application layer, which is used to provide end-user services and applications with which all stakeholders can interact (Stafford, 2015; Mohanraj et al., 2016; Hasanaj and Abuhemidan, 2019; Growing tomatoes from seed, 2022; Carbon Dioxide (CO2): Environmental Health in Minnesota, 2022; Quy et al., 2022). The stakeholders can connect with their mobile devices and web browsers to utilize the applications and services offered through the application layer. The second layer is the information management layer, which is responsible for data formation and categorization, creation, monitoring, and decision-making (Walter et al., 2017). The third layer is the network management layer, which includes technologies for communication such as GSM, Wi-Fi, 3G, UMTS, Bluetooth Low Energy, and ZigBee (Suanpang and Jamjuntr, 2019). Finally, the fourth layer is the information collection layer, which includes all sorts of physical IoT devices, such as sensors for sensing the environment and actuators for controlling and automating tasks (Sihombing et al., 2019; Montazeaud et al., 2021). We have noted that this architecture can be customized according to context and the needs of the solution, having more intermediate sublayers. Marcu et al. (2020) have introduced a SmartAgro telemetry system that can remotely monitor underlying crop conditions consisting of a local storage layer for processing the sensor data and an edge layer with decision-making capability for analyzing sensor data in real-time and filtering them without any latency.

In a typical smart farming setting, many sensors may be put in the agricultural field to measure different parameters, including humidity, soil pH, temperature, and light intensity (Walter et al., 2017). Each network device on the agriculture network is normally allocated a unique and distinct IP address for identification purposes. The IoT devices could be smart sensors, actuators, or wearable sensing devices (Mohammed et al., 2021). Once the data is collected, the collected data is delivered to the cloud or data analytic servers through a network gateway linked to the Internet by Wi-Fi or another communication media (Mohammed et al., 2021). Finally, the data is transferred from the cloud or data analytic servers to the farmer’s smart mobile devices or handheld computing devices, where they make informed decisions by examining this analyzed data.

As farmers are unable to be physically present in the field 24 hours a day and due to a probable lack of skills in employing various technologies to assess the appropriate environmental conditions for their crops, smart agriculture offers them automated solutions that can run without human supervision and can assist the farmers in making proper decisions to cope with the various types of challenges they may encounter. Further smart agriculture can contact and alert the farmer even when the farmer is not in the field, allowing the farmer to manage more acreage and increase productivity. With the overwhelming benefits that the farmers can now gain with the adoption of smart agriculture, many seek to adapt the technology as a viable alternative to eliminate the massive burden associated with traditional farming practices. In recent years, there has been a clear growth of research activities in smart agriculture. Lee et al. (2014) stated that IoT is a critical technology that will lead us to a sustainable world. They believe that IoT-powered smart agricultural solutions will change the status of the agriculture industry and business models, and farmers can profit more by adopting smart agriculture. By building a crop condition monitoring platform, Doshi et al. (2019) underlined that smart agriculture advancements have made traditional agricultural practices more flexible, cost-effective, and less wasteful. Ratnaparkhi et al. (2020) presented a review of various types of IoT agricultural sensors, which have been used in agriculture, and compared them with the different commercially available IoT sensors. With the conclusion remarks they derived, they stated that with the current rate of development of smart agriculture, if they are correctly used in countries like China, India, and Africa, it can easily end world hunger. Mekala and Viswanathan (2017) surveyed IoT-powered smart agriculture monitoring applications backed by cloud computing and highlighted its key enabling technologies. In their study, they have highlighted the necessity of an optimum smart agricultural architecture that is inexpensive, has low power consumption, able to provide good decision-making, and simple to comprehend for farmers who may lack the technological aptitude. Further they emphasized it would be an ideal solution for reaching out for more farmers and promoting smart agriculture. (Ayaz et al. (2019) discussed enabling technologies and platforms used in smart agriculture together with challenges currently faced by the industry and future prospects. In their study, they have emphasized that to benefit from every inch of farmland and to have a higher harvest, the adoption of smart agricultural practices is a must.

## 3 System design and implementation

After thoroughly investigating the most suitable plant to be grown in indoor lab conditions, the tomato plant has been chosen, as indoor lab environmental conditions best match with optimal conditions that tomato plants need (Schwarz et al., 2014; How to grow tomatoes indoors, 2022). Tomato, scientifically known as Solanum Lycopersicum (Schwarz et al., 2014; Walter et al., 2017), is considered an important horticultural crop worldwide due to its ability to grow in domesticated environments and is frequently used as a model crop for studying the development of fruits as well as for numerous cellular, molecular, and genetic investigations (Schwarz et al., 2014; Walter et al., 2017). Tomatoes can be easily grown in a controlled environment, such as in growth chambers or greenhouses, and require a daily light length between 8 to 16 hours to allow the plant to grow well, flower, and develop quality fruits. However, depending on the growth stage, the requirement for light may vary. Tomatoes also need a temperature of 10–35°C, relative humidity of 30–90%, and CO2 concentration of around 200–1500 ppm (parts per million) in an outdoor environment (Schwarz et al., 2014). Daily light conditions, CO2 concentration, and temperature all impact photosynthesis and biomass output (Walter et al., 2017), whilst temperature also controls the rate of phenological development. Tomatoes can be grown in soil, on substrates, or aeroponically without a substrate. Root volume and water uptake requirements are predominately determined by the transpiration demands of the plants; hence a good amount of wind is an essential factor for plants to grow in optimal conditions (Schwarz et al., 2014; Walter et al., 2017). The above-mentioned traits of tomatoes were best matched with our indoor university lab environmental conditions; thus, tomatoes were chosen as the main plant species for planting inside our indoor lab.

Having provided an overview of why we have chosen tomatoes to grow inside our indoor lab, we discuss the system design further in the next section.

### 3.1 System design

Our platform’s main objectives are real-time monitoring of environmental and soil conditions and managing irrigation and lighting conditions for our indoor tomato plantations. The proposed platform consists of the following components.

- IoT sensing devices that collect relevant soil and environmental parameters.
- Arduino Uno and NodeMCU microcontrollers to gather data from the IoT sensing devices, process and showcase the results, and stream the data to the cloud for further processing and analytics.
- Thinger.io is an open-source cloud platform for further data visualization, analytics, and actuator control.
- Grow light kit for helping the tomato plants grow under indoor environmental conditions by providing light, and irrigation system comprising of three peristaltic water pumps which can be controlled through the cloud.

**Figure 3:**
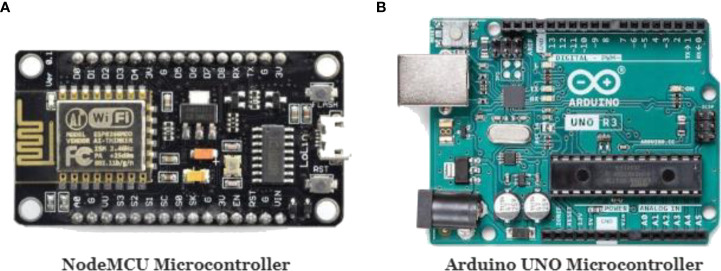
**(A)** NodeMCU microcontroller, **(B)** Arduino U.N.O. microcontroller.

The key component of our platform is the central NodeMCU microcontroller that streams data to the cloud through Wi-Fi and retrieves other sensing data from the Arduino Uno microcontroller through serial communication. NodeMCU is an open-source firmware and development kit that helps prototype IoT products within a few lines of code. It has 128 KB of RAM and 4 MB of flash memory for data storage and execution of programs (Kashyap et al., 2018; Parihar, 2019). Further, it also integrates with an 802.11b/g/n Wi-Fi transceiver, enabling the device to connect to a Wi-Fi network (Parihar, 2019). On the other hand, it can also set up a network on its own as the main node of a Wireless Sensor Network (WSN), allowing other devices to connect directly to it (Kashyap et al., 2018). **Figure 3A** depicts the NodeMCU microcontroller. Apart from the NodeMCU microcontroller, we have also used an Arduino UNO microcontroller, as most of the sensors we have used were analog sensors, and NodeMCU only contains one analog pin. In contrast, UNO contains multiple analog pins to attach more than one analog sensor, and we have connected three analog sensors to the UNO microcontroller.

Arduino UNO is an open-source microcontroller board based on the microchip AT-mega328P (Arduino, 2015). The microcontroller board has analog and digital input/output (I/O) pins that may be interfaced with various expansion boards and other circuits. Altogether it has 14 digital input/output pins, 6 analog inputs, a 16 MHz ceramic resonator, a USB connection, a power jack, an ICSP header, and a reset button (Badamasi, 2014). It contains everything needed to support the microcontroller, and the device can be powered by a USB cable or an external 9-volt battery, though it accepts voltages between 7 and 20 volts (Badamasi, 2014; Arduino, 2015). **Figure 3B** depicts the Arduino U.N.O. microcontroller.

**Figure 4:**
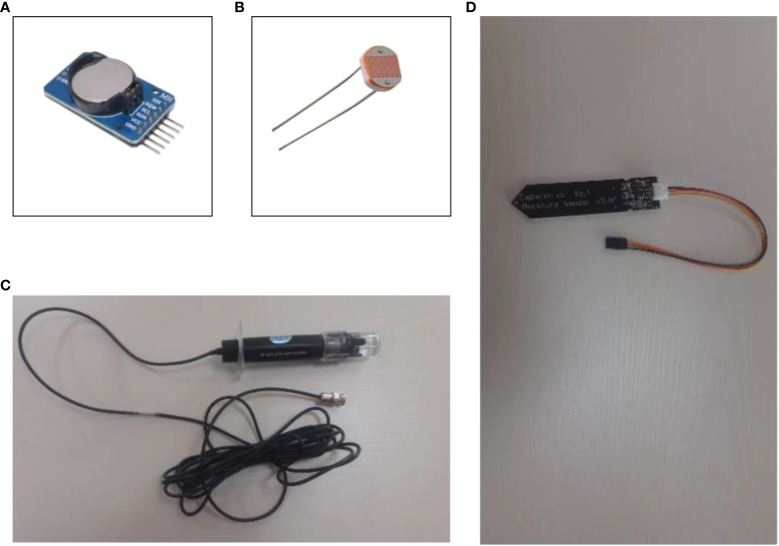
**(A)** DS1307 RTC module, **(B)** LDR, **(C)** H-101 analog pH sensor, **(D)** capacitive soil moisture sensor.

**Figure 5:**
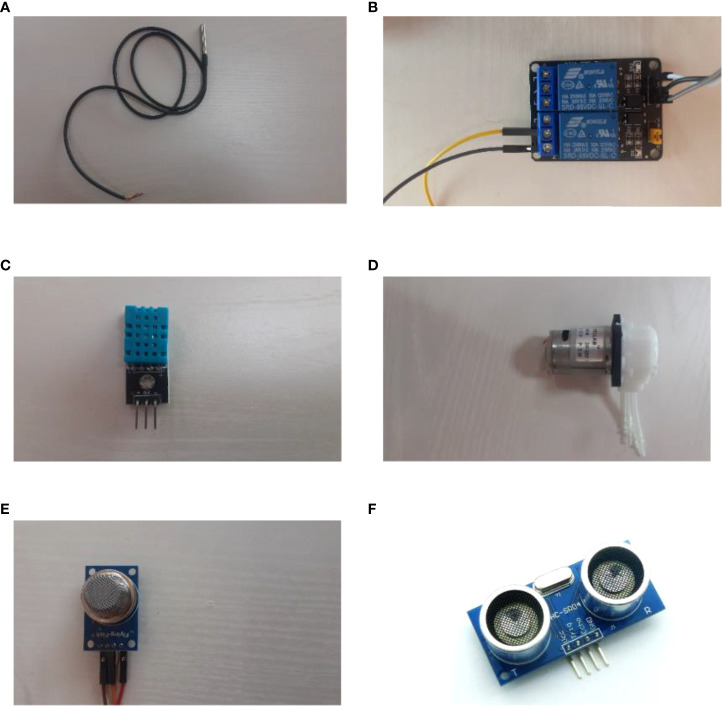
**(A)** DS18B20 sensor, **(B)** Two channel DC 5V relay module, **(C)** DHT-11 temperature and humidity sensor, **(D)** peristaltic pump, **(E)** MQ135 CO2 sensor, **(F)** ultrasonic sensor.

In terms of the IoT sensors, our platform accommodates eight sensors, a relay actuator, and three peristaltic pumps for the physical design of our platform. The sensors include a DHT-11 temperature and humidity sensor, H-101 analog pH sensor, capacitive soil moisture sensor, waterproof DS18B20 sensor, MQ135 CO2 sensor, and a light-dependent resistor (LDR), an ultrasonic sensor, and a DS1307 RTC (Real Time Clock) module. These sensors were chosen based on the parameters that needed to be measured whilst considering ease of use, economical value, and measurable parameters. **Figure 4** and **5** depicts all the different components, including sensors and actuators, utilized in the platform.

Components in **Figures 4** and **5** are explained in the following.

- **Figure 4A** shows the DS3231 RTC module, a real-time clock module that uses DS3231 IC as its backbone. It contains a backup battery mounted at the back of the module to keep track of time even in the absence of a main power source, with a chip capable of automatically switching between the main and backup power source as necessary (Suryawinata et al., 2017).
- **Figure 4B** shows an LDR sensor that works on the photoconductivity principle, with resistance varying according to light intensity. When the LDR connects with the 5V, it gives an analog voltage that varies depending on the input light intensity (Salim et al., 2015).
- **Figure 4C** depicts an industrial-grade analog pH sensor made from a sensitive glass membrane with low impedance. It can be used in pH measurements with fast response and excellent thermal stability, commonly used in aquaculture and water quality surveillance (Cucus and Febrianti, 2017).
- **Figure 4D** is a corrosion-resistant capacitive soil moisture sensor that outputs analog voltage based on capacitance changes (Eller and Denoth, 1996). As opposed to other resistive sensors, capacitive sensors do not require direct exposure to the metal electrodes, which can significantly reduce the erosion of the electrodes in the long run.
- **Figure 5A** shows the waterproof version of the DS18B20 temperature sensor that has an operating temperature range from −55 °C to +125 °C with a precision of ±0.5°C, and it operates within the range of 3.0V to 5.0V (Saha et al., 2021).
- **Figure 5B** shows the two-channel 5V relay module, which is used to control/switch high voltage (in our case, 12V) and current loads (Sunday et al., 2020).
- **Figure 5C** shows the DHT11 temperature and humidity sensor commonly used to monitor environmental temperature and relative humidity, and outputs the temperature and humidity values as serial data (Gay, 2018).
- **Figure 5D** shows the peristaltic pump, a positive displacement pump used for pumping various fluids. Three pumps have been used for the design of our automated irrigation system (Banerjee et al., 2017).
- **Figure 5E** showcases the MQ135 CO2 gas sensor, an air quality sensor for detecting a wide range of gases, including NH3, NOx, alcohol, benzene, smoke, and CO2 (Abbas et al., 2020). For our platform, we have used MQ135 to measure CO2 concentration in ppm.
- **Figure 5F** shows the ultrasonic sensor, which measures the distance to an object using ultrasonic sound waves with high reliability (Latha et al., 2016).

**Table 1** summarizes the device specification of IoT sensing devices we have used along with their purpose.

**Table 1:** Specifications and the purpose of IoT devices.

IoT sensor	Specifications	Purpose
DS1307 RTC module *(Suryawinata et al.,2017)*	•Operating voltage: 3.3V – 5V •Battery type: LIR2032 rechargeable lithium battery •The real-time clock provides hours, minutes, seconds, and AM/PM.	To maintain timely information when streaming data to the cloud
LDR sensor *(Salim et al.,2015)*	•Operating voltage: 3.3V or 5V •Operating current: 15ma	To get the amount of light falling on a surface
H-101 analog PH sensor *(Cucus and Febrianti,2017)*	•Operating voltage: 5.00V •Measuring range: 0-14 PH. •Measuring temperature: 0-60 °C •Accuracy: ± 0.1pH (25 °C) •Response Time: ≤ 1min	To Measure the Soil PH.
Capacitive soil moisture sensor *(Eller and Denoth,1996)*	•Operating voltage: 3.3 V to 5.5 V •Operating current: 5mA. •Analog output. •Weight (gm): 15	To measure the soil moisture percentage
DS18B20 sensor *(Saha et al.,2021)*	•Usable temperature range: -55 to 125°C • ± 0.5°C accuracy from -10°C to +85°C •Usable with 3.0V to 5.5V	To measure the soil temperature
Two-channel DC 5V relay module *(Sunday et al.,2020)*	•Voltage to operate: 5V •Load: 10A, AC 250V/15A, 125V	To control our irrigation water pumps and grow light
DHT-11 temperature and humidity sensor *(Sunday et al.,2020)*	•Operating voltage: 3.5V to 5.5V •Operating current: 0.3mA (measuring) 60uA (standby) •Temperature Range: 0°C to 50°C •Humidity Range: 20% to 90% •Accuracy: ± 1°C and ±1%	To get the environmental temperature and relative humidity
Peristaltic pump *(Banerjee et al.,2017)*	•Working Temperature: 0°C - 40 °C •Motor voltage: 12V. •Motor current: 200-300mA. •Flow rate: up to 100 mL/min.	Provide water to the plants
MQ135 CO2 sensor *(Abbas et al.,2020)*	•Operating voltage: 5V •Detects NH3, NOx, alcohol, Benzene, smoke, CO2, etc. •Analog output voltage: 0V to 5V •Preheat duration: 20 seconds	To measure the CO2 concertation in an indoor environment
Ultrasonic sensor *(Latha et al.,2016)*	•Operating voltage: 3.3V – 5V. •Operating Current: 8mA. •Working Frequency: 40Hz. •Ranging Distance: 3cm – 350cm/3.5m. •Measuring Angle: 15 degrees.	To measure the height of the plants

**Figure 6:**
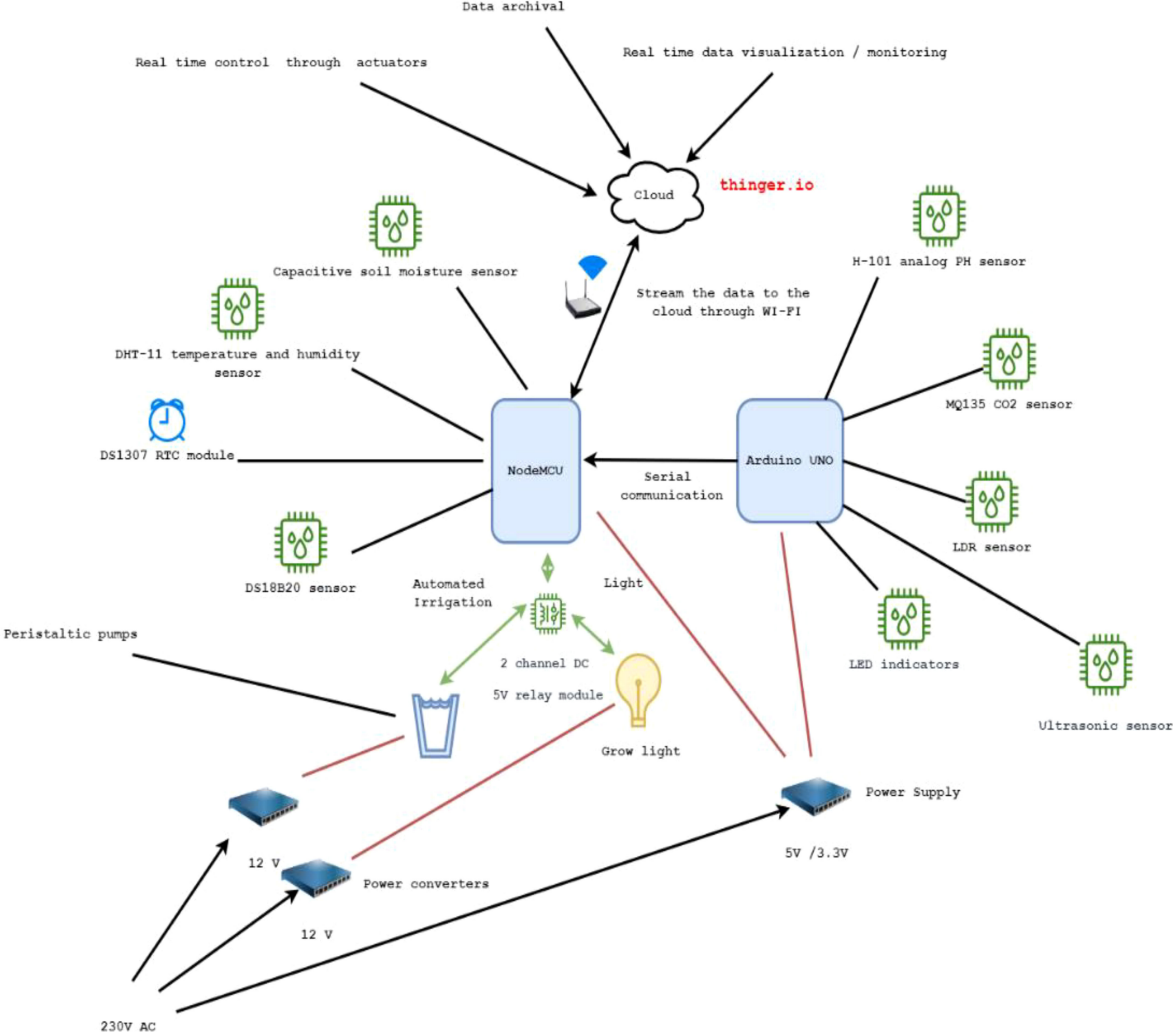
Block diagram of our platform with all the connections.

Having provided a brief technical overview of each of the physical components in the platform, the next steps involve designing the platform with correct wiring and connections. **Figure 6** below shows the block diagram of our platform with all connections.

The pH sensor, CO2 sensor, LDR sensor, LED indicators, and Ultrasonic sensor were attached to the Arduino UNO microcontroller, whilst the capacitive soil moisture sensor, DHT-11 sensor, DS1820 module, and RTC module were attached to the NodeMCU. A serial communication gateway between UNO and NodeMCU was also opened to allow the UNO to send sensor data to the NodeMCU for dispatch the data to the cloud via its Wi-Fi connection.

**Figure 7:**
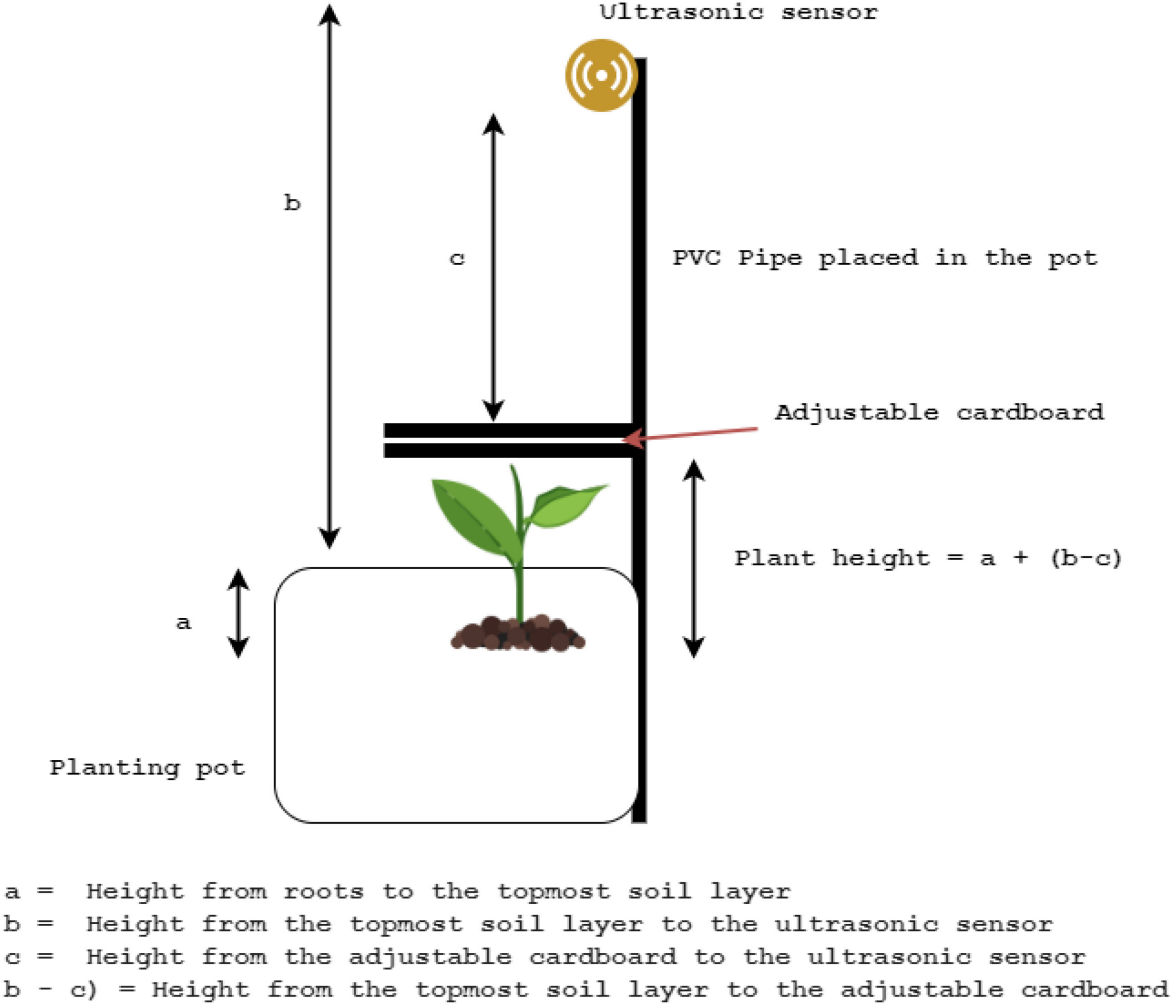
Measure the plant height through ultrasonic sensor.

**Figure 8:**
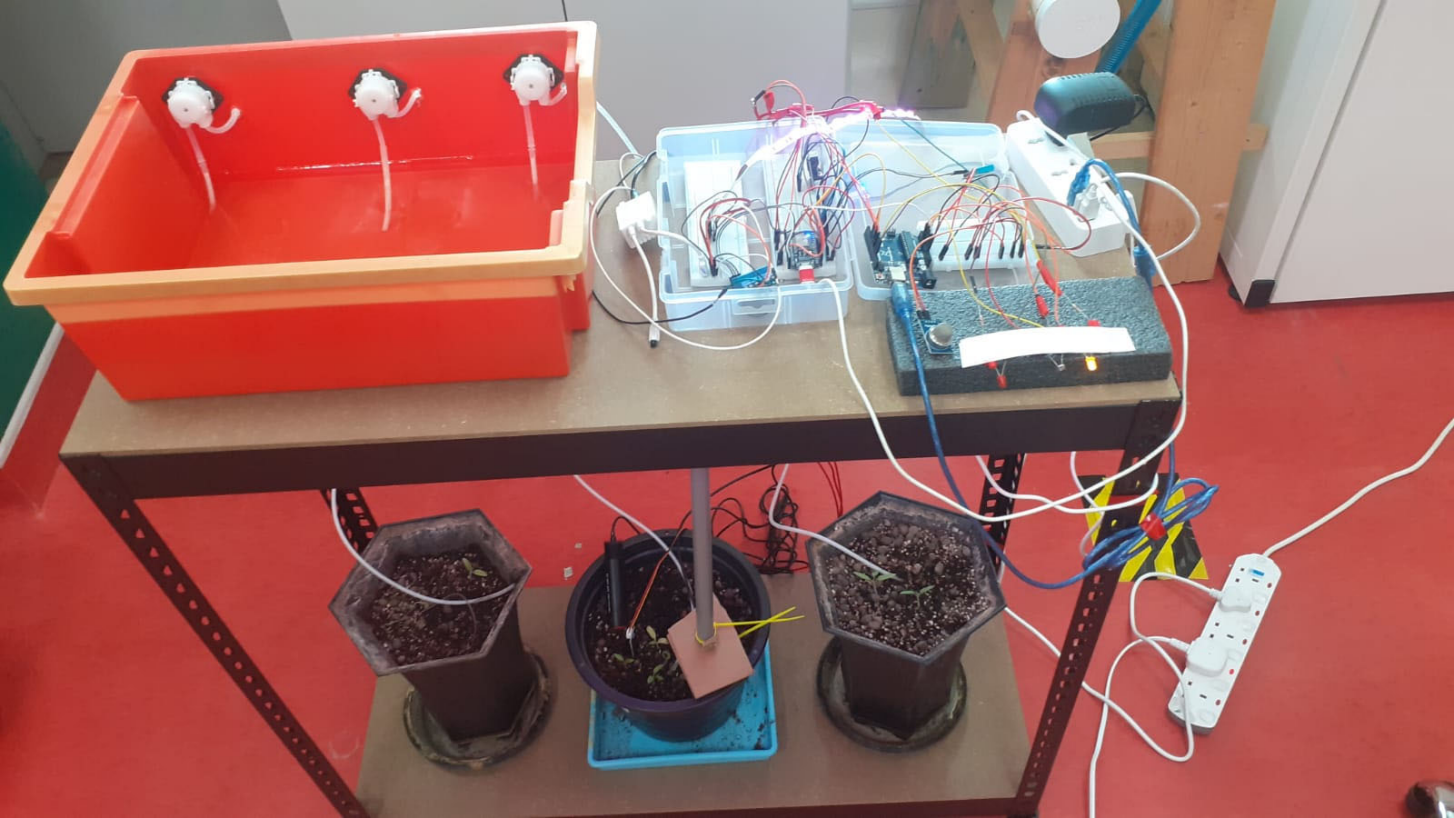
The physical design of the platform.

The two-channel direct current 5V relay module was used to control and automate the grow light kit and irrigation system, enabling control of them over the cloud. Three water pumps were parallelly connected, where one connection end was connected with the relay, whilst the other end of the relay was connected to the grow light kit. pH sensor, CO2 sensor, capacitive soil moisture sensor, and all remaining sensors were tested for their measuring accuracy. The ultrasonic sensor was attached to the top of a PVC pipe set up, as shown in **Figure 7**, and was used to measure the height of the plant as an indication of plant growth. Manual daily measurement of the plant was also performed as a supplement to automatic measurement using the ultrasonic sensor. A Wi-Fi connection was used to stream sensor data to the cloud every five seconds, as well as to receive data from the cloud. We have decided to use a time interval of five seconds in order to see the real-time variations of sensor data, as well as to facilitate the real-time control of actuators with a quick response time. **Figure 8** showcases our platform’s physical design with all the wiring connections.

**Figure 9:**
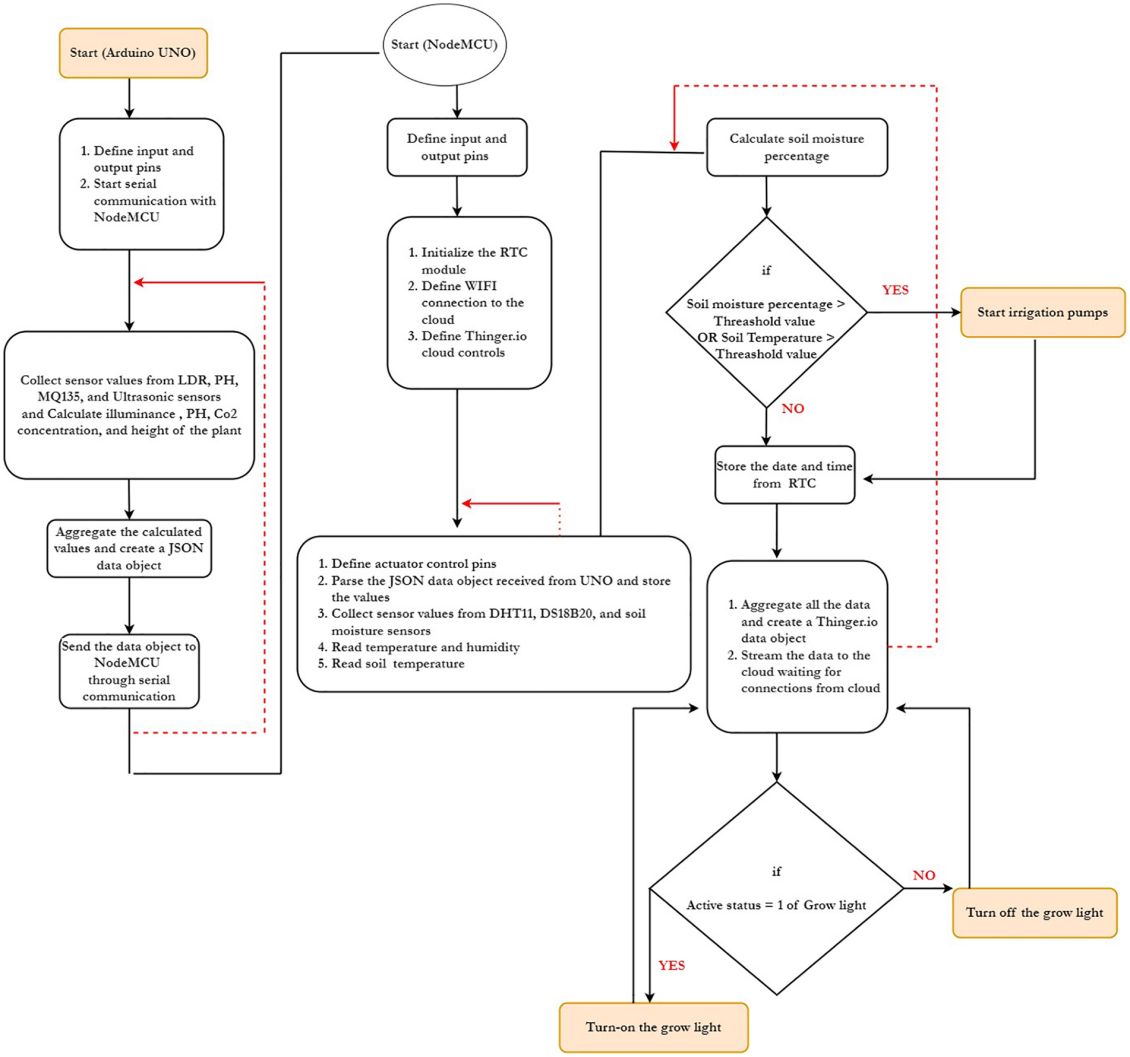
The workflow of our platform.

Arduino Integrated Development Environment (IDE), written in Java programming language and C/C++ programming language, was used for programming the setup. Program sketches from the Arduino IDE were then compiled, debugged, and uploaded to both NodeMCU and UNO microcontrollers for real-time execution. **Figure 9** shows the workflow of our platform step by step until streaming data to the loud.

### 3.2 Configuring connections to the cloud

As illustrated in **Figure 6**, once the data are collected from both the UNO and NodeMCU microcontrollers, the NodeMCU is responsible for streaming the data to the cloud every five seconds. To fuse the heterogeneous data coming from a variety of IoT sensors and process them in the cloud in real-time, the Thinger.io platform, which is an open-source platform having the capabilities for collection, management, and analysis of vast amounts of heterogeneous IoT sensor data in the cloud (Luis Bustamante et al., 2019; Suciu et al., 2019; O’Grady et al., 2019; Trilles et al., 2020; Thinger.io, 2022), was used.

**Figure 10:**
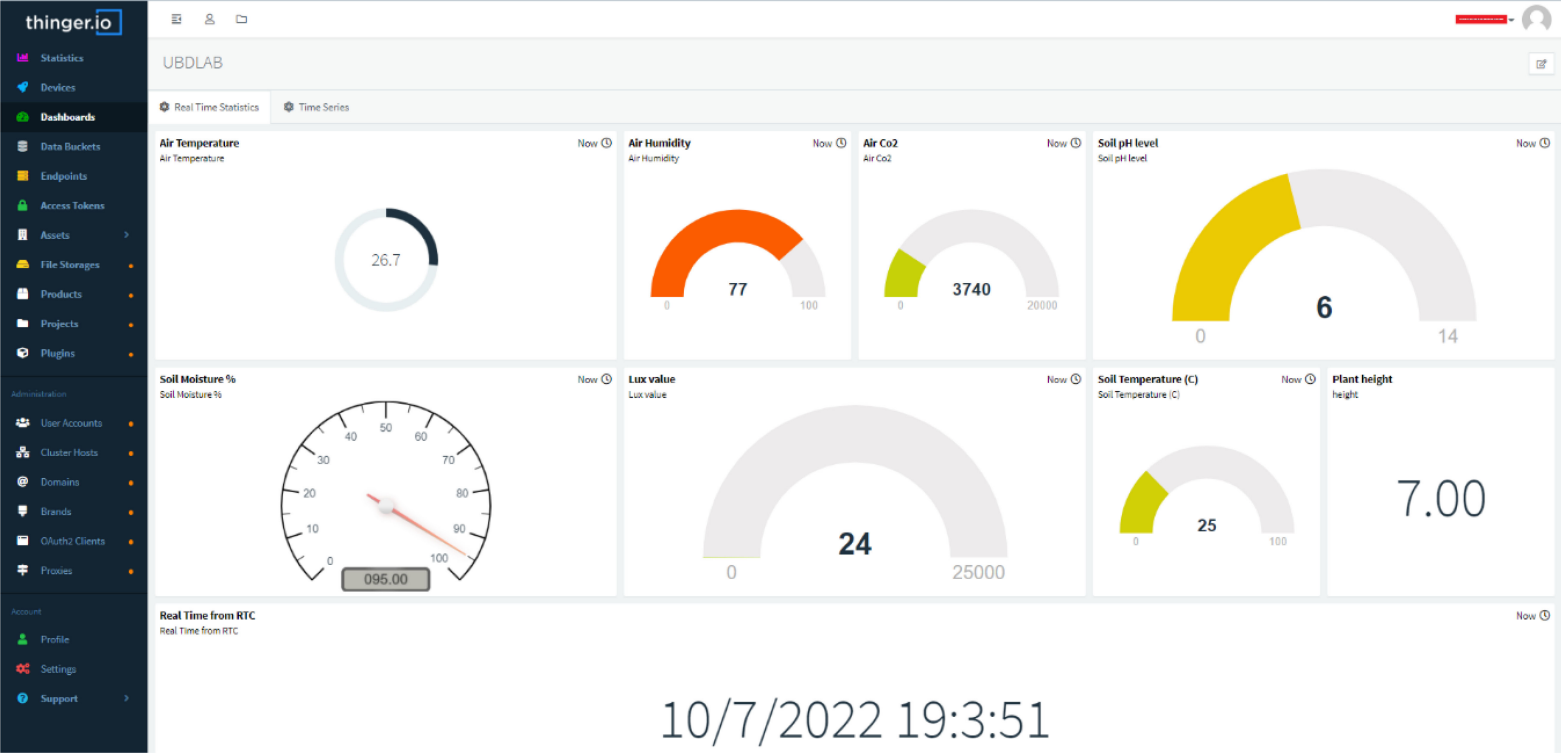
Cloud dashboard made for data visualization and actuator control in Thinger.io.

To offer a brief overview of Thinger.io, it provides a free and open-source cloud solution that allows the simple and easy implementation of data fusion IoT applications in the cloud (Luis Bustamante et al., 2019). Moreover, it offers a free tier service for connecting a limited number of IoT devices, thereby allowing remote sensing and actuation (Thinger.io, 2022). Thinger.io also facilitates the installation of the software outside the cloud for private and personal use, allowing users to create their customized platforms as opposed to other available solutions. Different Internet-enabled devices, including Arduino, Raspberry Pi, NodeMCU, and ARM devices, can be connected to the platform. It also provides a web-based dashboard for remote monitoring and management of all resources (Luis Bustamante et al, 2019; Thinger.io, 2022). Considering the feasibility, free tier services, and flexibility, Thinger.io has chosen for data visualization, analytics, and actuator control over the cloud. **Figure 10** illustrates the cloud dashboard that has made on Thinger.io for visualization of our sensing data and actuator control , based on the received data to the cloud. For each sensing parameter, a separate display widget was created on the dashboard, and another widget was created to visualize the correct timestamp, as seen in **Figure 10**. Two ON/OFF control buttons were also added to automate grow light kit and the irrigation system.

## 4 Experimental results and discussion

After the platform had been set up, tomato plants were grown inside the lab. The growing of tomato plants requires adequate ventilation and light. The lab was fully air-conditioned and did not have enough windows for proper ventilation. According to the stages of tomato plant growth; tomato plants grow in several stages: 1) the germination and early growth with initial leaves, which is between 25 and 35 days, 2) the vegetative period, which is between 20 to 25 days, 3) the flowering period, which is between 20 to 30 days, 4) an early fruiting period, which is between 20 to 30 days, and 5) mature fruiting stage which is between 15 to 20 days (Max et al., 2009; Leyva et al., 2015). However, according to the research (Max et al., 2009; Leyva et al., 2015; Shamshiri et al., 2018), it is evident that the exact date of each stage depends on varieties and other environmental factors, such as soil condition, air temperature, nutrients, and light.

**Figure 11:**
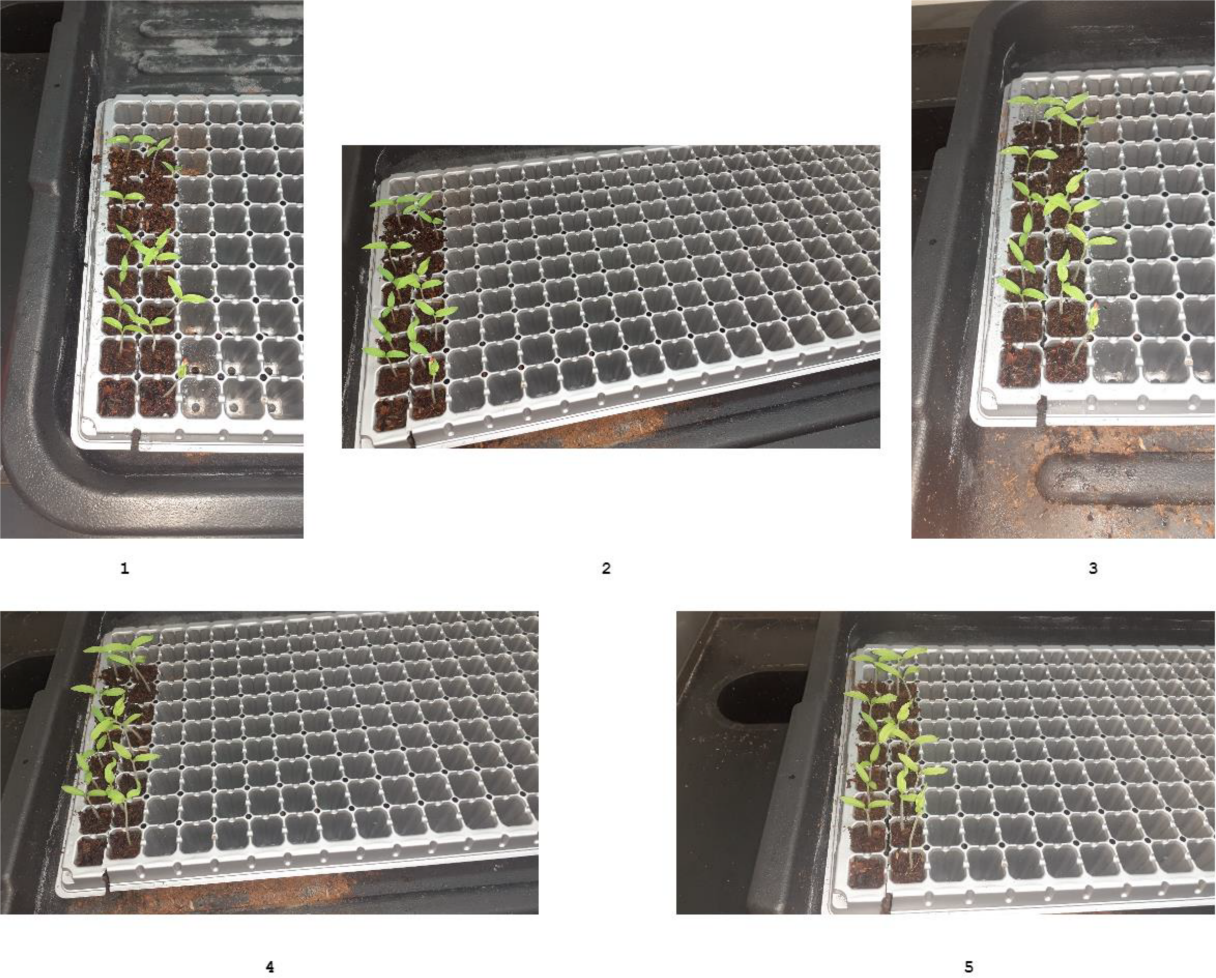
Germination stage of our tomato plants (1. 10^th^ day 2. 11^th^ day 3. 12^th^ day 4. 13^th^ day 5. 14^th^ day).

The seeds were seeded in the planting trays and kept for the initial germination phase of around 15 days. **Figure 11** shows the germination stage of our plants inside the lab environment step by step in sequential order.

**Figure 12:**
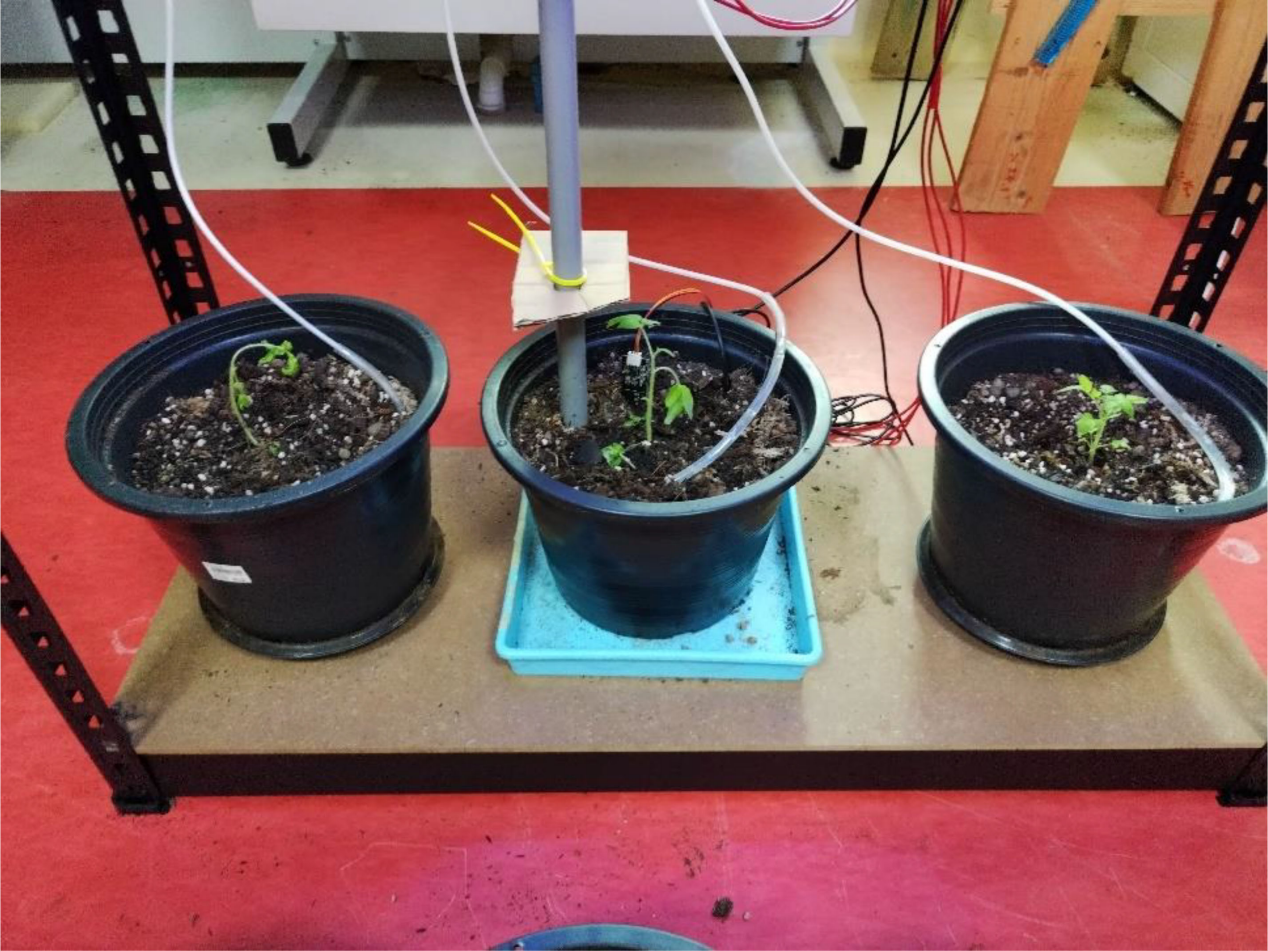
Growing tomato plants inside lab environment with sensors deployed in soil.

After completion of the germination stage, the healthy grown plants were moved to three planting pots, where the platform with sensors was deployed to capture data on the underlying soil and environmental conditions, as shown in **Figure 12**. The central pot was the main tomato plant, whilst plants in the remaining pots were used as a replacement in case something went wrong with the main plant and as a validation setup for our experiment. Soil moisture, pH, soil temperature, and ultrasonic sensors were embedded in the central pot where we have our main plant, with the remaining sensors set up on the top of the planting rack. Calibration and initial testing were done on all the sensors prior to deployment.

**Figure 13:**
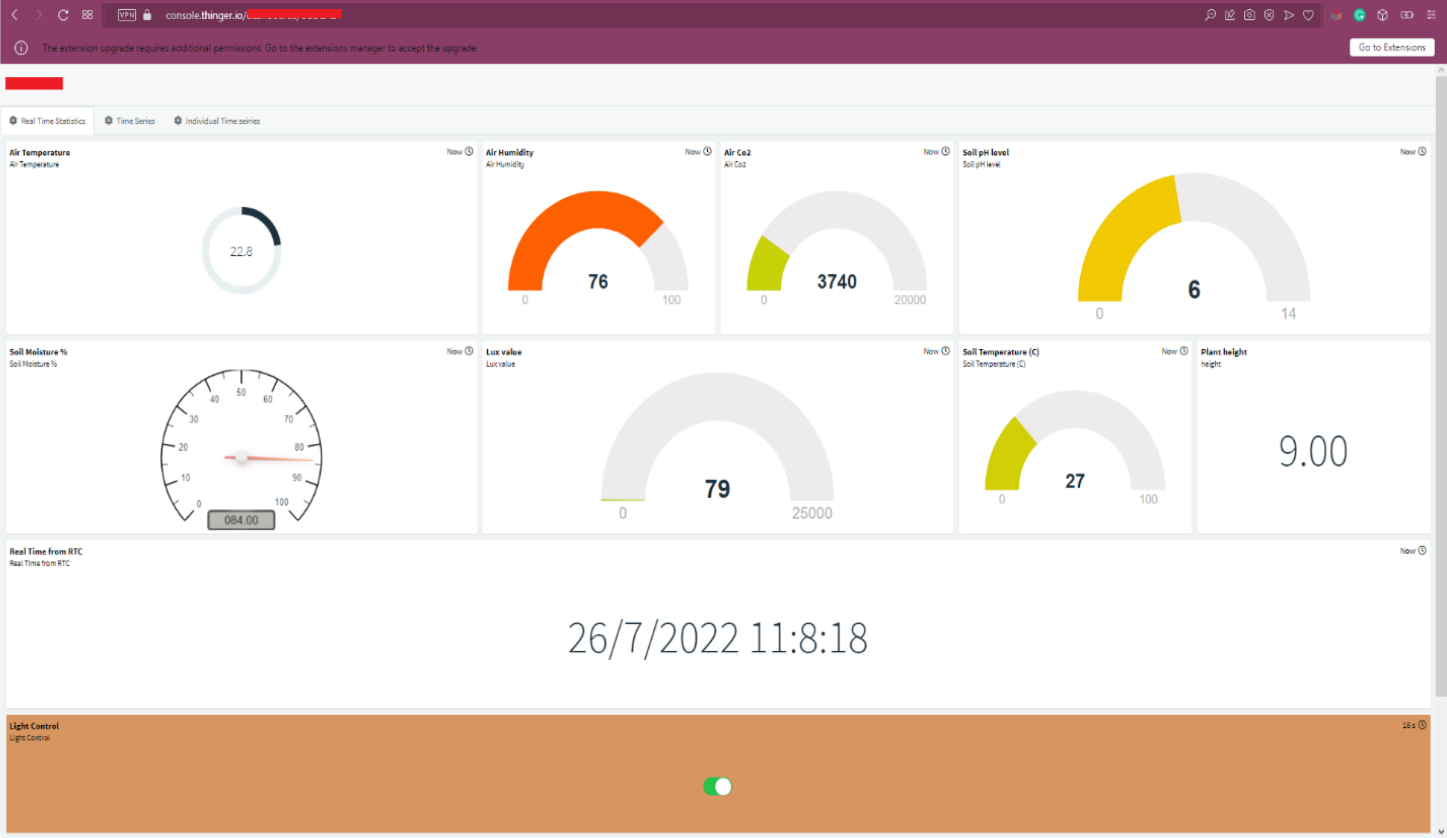
Real-time dashboard.

**Figure 14:**
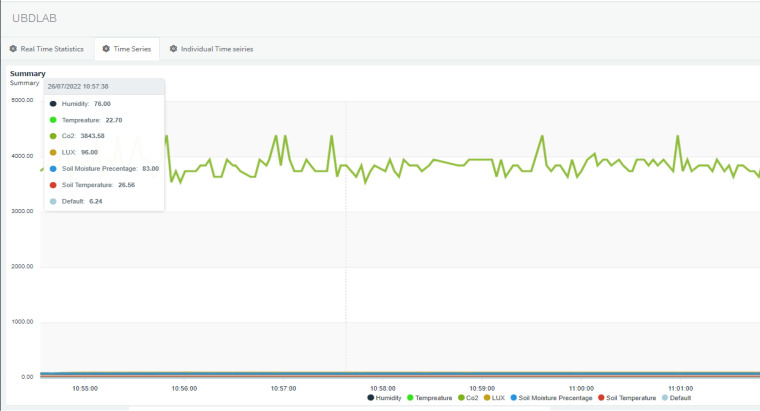
Time series data.

**Figure 13** depicts the shareable real-time dashboard for visualizing real-time data streamed from the NodeMCU microcontroller. Separate widgets for displaying real-time sensor data: air temperature, air humidity, CO2 concentration, soil pH, soil moisture percentage, lux value, soil temperature, and plant height as well as stamped time from the RTC module, are made available on the dashboard. Using stamped time from the RTC module allows the real-time sensor values to be archived with the correct timestamp. An ON/OFF control button to control the grow light kit and the irrigation system was also added to the dashboard. Another tab had also been set up in the same dashboard for visualizing real-time and archived time series sensor data, as shown in **Figure 14**, to give more insights on the changes in sensor values.

**Figure 15:**
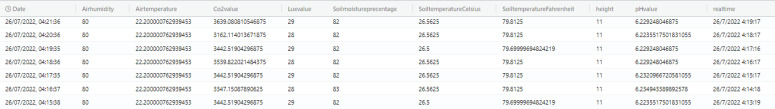
Archived sensing data in a data bucket.

For the data archiving, data bucket functionality in Thinger.io was used. The data bucket functionality allows virtual data storage of time series information, such as the sensor data gathered over time (Luis Bustamante et al., 2019), as shown in **Figure 15**. Once the data is archived through the data bucket, it can also be exported in CSV or JSON format for further analysis.

**Table 2** shows the statistical summary of the gathered sensor data through our cloud platform, between 31st of July 2022 at 12.00 AM to 7th August 2022 at 12.00 AM, containing over 10000 data points.

**Table 2:** Statistical summary of gathered data.

	Relative Humidity	Air temperature	Co2value	Lux value	Soil moisture percentage	Soil temperature	pH value
**Average**	78.38	22.43	2278.80	51.49	80.19	26.52	5.68
**Standard** **Deviation**	2.12	0.66	440.24	34.51	6.82	0.55	0.74
**Minimum**	72.00	20.80	1470.00	19.00	71.35	25.68	5.61
**Maximum**	84.00	25.40	2998.00	112	98.00	28.43	5.75

**Figure 16:**
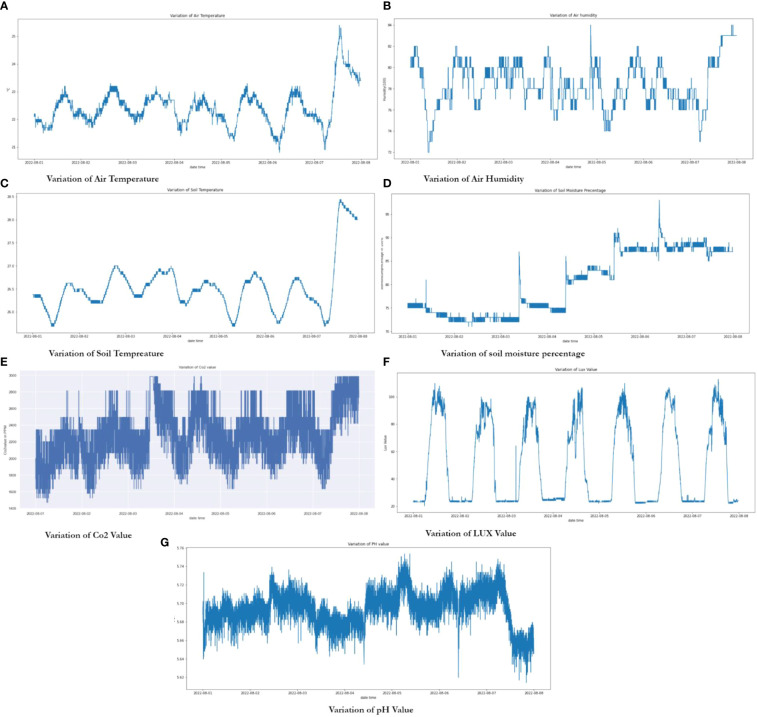
Variation of environmental and soil parameters; **(A)** Variation of Air Temperature, **(B)** Variation of Air Humidity, **(C)** Variation of Soil Temperature, **(D)** Variation of soil moisture percentage, **(E)** Variation of Co2 value , **(F)** Variation of LUX value , **(G)** Variation of pH Value.

**Figures 16A-G** show air temperature, humidity, soil temperature, soil moisture, CO2, light intensity, and pH variations, respectively, during the observation period. Generally, temperature slightly increased over the period, reaching a top of nearly 26°C inside the lab environment, with rising and reduced temperature during the daytime and nighttime, respectively. It is noted that temperature remained within the optimal range (10–35°C) that plants require throughout the period, as seen in **Figure 16A**. **Figure 16B** shows the variation of relative humidity. As the relationship between temperature and humidity are inversely proportional to each other, temperature increases would correspond to a reduction in humidity, which can be verified from **Figures 16A, B**. Similar to temperature, humidity also remains within the optimum range of 30 to 90 during the period.

As can be seen from (**Figures 16C and A**), the soil temperature variation is much similar to the air temperature variation. Tomato plants generally prefer warmer weather if moved (transplanted) or planted outside. Hence the soil temperature at night should not reduce to below 13°C (Growing tomatoes from seed, 2022), such that plants would not experience too low of a temperature. A low-temperature environment would result in growth retardation, which would eventually lead to poor fruit production or even plant mortality. However, data from our platform indicate that soil temperature consistently stays above the minimum temperature requirement throughout the period.

The soil was watered before planting the tomato plant in the pots, and consequently, the soil moisture percentage gave a reading of more than 75. Afterward, as seen in **Figure 16D**, the soil moisture percentage has slightly reduced, and by physical observation and with the observed real-time data from our dashboard, we activated the irrigation pumps through the cloud platform for approximately 90 seconds which was predetermined while testing our irrigation system for how much duration we need to supply water. Afterward, as seen in **Figure 16D**, the value drastically increased, which can be seen as a sudden spike. Over the period, we have activated the pump six times by relying on our real-time data on the dashboard.

CO2 concentration inside the lab varied between 1470 to 2988 ppm during the observation period, as can be seen from **Figure 16**(e). A lab or room with a good air exchange should have CO2 concentration readings within the range of 350-1,000 ppm (Suanpang and Jamjuntr, 2019; Carbon Dioxide (CO2): Environmental Health in Minnesota, 2022), where inadequate ventilation may lead to the accumulation of many pollutants inside. In such case the indoor carbon dioxide (CO2) level can be used as a yardstick for a analyzing the room’s ventilation level. (Hasanaj and Abuhemidan, 2019; Carbon Dioxide (CO2): Environmental Health in Minnesota, 2022). It can be seen that the lab environment has slightly higher CO2 concentration readings as the lab does not have adequate ventilation. This is similar to references (Hasanaj and Abuhemidan, 2019) and (Aminulloh et al., 2019), whereby CO2 concentration readings of over 4000 ppm over three days in a room with no proper ventilation, and CO2 concentration readings of over 4000 ppm in a Greenhouse environment, respectively, had been observed. Over the observation period, it can be observed that CO2 concentration readings fluctuated between 1600 to 2400 ppm. The recommended CO2 concentration readings for tomatoes after transplanting are between 800 to 1000 ppm in a typical outdoor setting, and it can be clearly seen that CO2 concentration readings obtained slightly deviated from the recommended values despite the plants growing considerably well (Hasanaj and Abuhemidan, 2019). This can be mainly attributed to the inadequate ventilation in the room.

In **Figure 16F**, it can be seen that light intensity decreases during the nighttime whereas increasing during the daytime, with values ranging between 19 to 112 Lux. The activation of grow light kit doesn’t impact the light intensity we measured as we only activated the grow light during the daytime between 8.00 AM to 7.00 PM as a replacement for sunlight. Further, we have configured the LDR on the top of the planting rack, not near the plants, as we can measure how the light intensity varies inside the lab environment.

pH variation over the observation period is depicted in **Figure 16G**, which clearly shows a slight fluctuation of the pH in between the range of 5.6 to 5.8. According to the optimum pH range that tomato plants can bear, which is between 5.5 to 6.8, it is evident that tomatoes can tolerate slightly acidic soils down to a pH of 5.5, but for the best harvest, it should be between 6.0 to 6.5 pH. On the other hand, tomatoes are an acid-loving plant that is best grown in soils with a pH below 7.0 (Kagita et al., 2021; Quy et al., 2022; Thilakarathne et al., 2022), which we can clearly conclude soil pH conditions are optimum for our plants to grow well.

## 5 Discussion

Based on the analysis of the collected sensor data and visual observation of the plants from the biological perspective (e.g.: height of the plants and number of secondary leaves), it is evident that our tomato plants are growing well and healthy in the indoor lab environment, with the plant height increasing almost 2 cm during the period that the proposed platform was deployed. However, more tests are required to further determine the data validity and operability of the current system before the system can be scaled up and deployed on a real plantation. It is noted that the designed prototype platform offers greater benefits for farmers with a simple IoT setup and open-source technologies. Additionally, the platform has been built with cost-effective IoT devices, which cost less than 70 USD. **Table 3** gives the costing of the proposed platform.

**Table 3:** Amount spent on IoT devices and enabling technologies.

**IoT devices/enabling technologies used**	**Spend cost in USD**
NodeMCU microcontroller	2
Arduino UNO microcontroller	3.5
DS1307 RTC module	0.5
LDR sensor	0.05
H-101 analog PH sensor	40
Capacitive soil moisture sensor	0.5
DS18B20 sensor	1
Two-channel DC 5V relay module	1.5
DHT-11 temperature and humidity sensor	1.7
Three peristaltic pumps	10
MQ135 CO2 sensor	1.5
Ultrasonic sensor	1.5
Cloud for data visualization/archival and actuator control	Free
**Total cost incurred**	**63.75**

The platform has been designed as a low-cost smart agricultural solution, especially for indoor environments, with a special focus on urban farming by considering time saving, space, cost, and convenience, which are important considerations for urban agriculture. Overall, throughout the observation period, the platform has been demonstrated to be stable in terms of reliability and operability, with no malfunctioning of devices or loss of data. However, the prototype system does lack some capabilities and may require further upgrades and expansions, including the capability of visually monitoring the plants.

## 6 A comparison between some similar works

To better compare our work with similar research, in **Table 4**, we compare our work with theirs in terms of available features and components used.

**Table 4:** Comparison between similar work.

Reference	Microcontroller	Connection	Platform	Real-time	Cloud-enabled	Actuator control	Data archival and download for further analytics	Allows crop management	Development and implementation cost	Sensors	Phenomenon
(Bates et al., 2021)	A LoRaWAN-enabled node	LoRaWAN	FarmDecisionTECH	✓	✓	x	x	✓	High	Temperature, Salinity	The researchers have designed an aquaculture-based environmental monitoring network consisting of LoRaWAN-enabled atmospheric and marine sensors attached to buoys on Clyde River, Australia, to make precise and rapid decisions for oyster farmers operating in the river to notify them against adverse environmental threats.
(Forcén-Muñoz et al., 2021)	Wireless data logger	Wi-Fi	Irriman	✓	✓	x	x	x	High	Drill and Drop, Water potential sensor, Flow meter	The authors describe a cloud-based platform capable of acquiring data from a wide range of agronomic sensors for crop monitoring and irrigation management to assist agronomists in optimizing irrigation procedures *via* a usable web-based tool that allows them to elaborate irrigation plans and evaluate their effectiveness over crops.
(Mohammed et al., 2021)	NodeMCU	Wi-Fi	Thing-Speak	✓	✓	✓	x	x	Intermediate	Temperature, humidity, Flow meter,Anemometer,Soil moisture	The authors have designed a fully automated controlled subsurface irrigation system to control a modern subsurface irrigation system for improving irrigation management of date palms in arid regions.
(Herman et al.,2019)	NodeMCU	Wi-Fi	ThingSpeak	✓	x	✓	x	✓	Low	PH sensor, Total Dissolved Solids (TDS) sensor	The researchers demonstrated a smart agriculture system that measures pH, TDS, and nutrient temperature values in the nutrient film technique (NFT) technique using IoT sensors to predict the nutrient conditions where they used the predicted results to provide commands to microcontrollers for switching the state of the nutrient controller.
(Mehra et al., 2018)	Arduino U.N.O., Raspberry Pi3	Wi-Fi	–	x	x	✓	x	✓	Intermediate	Temperature, Humidity, Water level sensor, Photo resistor, PH sensor	The authors created an IoT-based hydroponic system that uses Deep Neural Networks to give the necessary control action for the hydroponic environment based on several input factors.
(Mohanraj et al.,2016)	Arduino U.N.O.	Cable Internet	Blynk	✓	✓	x	x	x	Low	Temperature, Humidity sensor, Soil moisture sensor, Magnetic float sensor, BH1750 Module	The authors proposed an e-Agriculture application with the intention of designing a knowledge management platform for farmers.
(Suciu et al.,2019)	Raspberry Pi	Wi-Fi	Grafana	✓	✓	x	x	x	Intermediate	–	The authors have demonstrated how agriculture big data and decentralized cloud operations are happening; by designing a secure cloud monitoring framework for smart agriculture.
(Trilles et al.,2020)	Linkit One	GPRS	SEnviro	✓	x	x	x	✓	Intermediate	Temperature and Humidity, Rainfall sensor	The authors created an IoT platform capable of monitoring weather events to predict diseases and alert farmers in a vineyard.
(Aminulloh et al.,2019)	NodeMCU.	Wi-Fi	–	x	x	x	✓	x	Intermediate	Temperature and Humidity, TSL2591, CCS881 sensor	The authors have designed an offline IoT platform for feature extraction and to monitor the development of tomato plantations in a greenhouse environment.
Our work	NodeMCU. Arduino U.N.O.	Wi-Fi	Thinger.io	✓	✓	✓	✓	✓	Low	Temperature, Humidity, PH sensor, Ultrasonic sensor, RTC module, Soil moisture sensor, LDR, DS18B20, MQ 135 Co2 sensor	Created a real-time, low-cost crop management platform for the management of indoor plantations; especially for urban farming (e.g., vertical farming)

**Table 4** compares other works in the literature with our work in terms of smart agricultural platforms designed. The following characteristics have been offered to describe each one:

Microcontroller – refers to the microcontrollers model that the research uses
Connection- refers to the wireless or wired connection available.
Platform – indicate whether the research uses a specific platform and its name.
Realtime – shows whether the system works in real-time or not.
Cloud-enabled – suggest that the research uses cloud computing services.
Actuator control -indicates whether the devices in the system can be controlled depending on the situation.
Data archival and download for further analytics – indicate whether the system facilitates to archival of the time series data and allows the users to refer to them at a later time.
Allows crop management- indicate whether the system facilitates real-time monitoring and control.
Development and implementation cost – describe whether the overall development and implementation cost is high, intermediate, or low.

Based on the above, it is evident that most works have used Arduino as the main microcontroller and depended on Wi-Fi for their communication needs, with a cloud platform mostly utilized to perform real-time monitoring and actuator control. In terms of the sensing devices, a total of nine sensors have been utilized in our research for sensing the ambient environment as opposed to other research, which only used a limited number of sensors. Despite this, the total costs of the proposed platform is almost similar to some of the works found in the literature. Further, with the results we obtained, it is noted that the system is able to provide accurate results in real-time, providing us the opportunity to monitor the underlying crop conditions in real-time and control certain parameters. Apart from the Co2 concentration, we have obtained all other parameters in a manual way, and it is noted that all the values are almost accurate. On the other hand, during the time that the system is active, no functional anomalies were detected which proved the reliability of the system.

## 7 Future work

The main intention of this section is to provide our readers with a brief understanding of how our proposed work can be extended toward indoor plant condition monitoring and urban farming. With the proposed work in the research, we have proved that our system can be built using low-cost IoT sensors and is capable of providing reliable service as long as the power and Internet connection are stable. Regarding indoor and urban farming, resources such as water and nutrient-rich soil are very limited, and the growers engaged in such farming have a limited time to spend. Such indoor and urban farming examples entail gardening, vertical farming, and soilless farming methods such as hydroponics, aeroponics, and aquaponics. According to the literature, it is evident that there is only less research work done pertaining to the use of smart agricultural solutions for indoor and urban soil-based farming (Khattab et al, 2016). On the other hand, we have noted with regard to using smart agricultural solutions towards indoor and urban farming, a lot of research on soilless farming (Patil et al., 2020 ; Charumathi et al., 2017 ; Kour et al., 2022).

Due to various factors such as inflation, supply chain interruptions, natural disasters, the COVID-19 pandemic, and environmental pollution dealing with agriculture and food insecurity concerns, many people living in urban areas are now keen on producing their own food at home. This has become a common trend, and progressively within time, by the next two decades urban and indoor farming would be much more popular in urban areas. We have developed our platform, having all these things kept in our mind, and our platform can be deployed in such a way, as to monitor the condition of a particular chosen plant in the plantation and, based on that, provide insights pertaining to the underlying plantations as it is not feasible to deploy such IoT solutions throughout the plantation. With a simple maneuver, by replacing only the necessary sensors pertaining to the monitoring parameters, our proposed platform can be used to monitor the conditions for soil less-farming such as with hydroponics, aeroponics, and aquaponics.

As per the next steps of our work, we have planned to integrate a real-time camera for real-time visualization of plants and integrate an intermediate Raspberry Pi device as an edge device for accumulating sensor data coming from NodeMCU to analyze our data in the edge of the network and provide real-time notifications/alerts based on the underlying conditions in near real-time for farmers. Further, with the introduction of this edge device and incorporating it with AI, we plan to provide total autonomy to our system as with the data analytics at the network edge, the system would be able to learn from the data and would be able to automate irrigation and lighting for plants with no sort of intervention from the cloud or farmers. Thus, this would be an ideal solution for indoor and urban farming for the optimum utilization of resources. In this work, we have used all low-cost sensors and open-source solutions, and we believe this would pave the way for the design of low-cost but effective and reliable smart agriculture solutions, which would become much more popular among farmers with low income.

## 8 Conclusions

The main objective of this research is to design and implement a flexible cloud-enabled low-cost sensorized IoT platform for real-time monitoring and automating tasks dealing with indoor plantation. In this regard, we have chosen tomatoes to be planted in the lab environment and to deploy our platform to check the functionalities and reliability of our platform with the crop we have chosen. Through the platform, we have visualized real-time data and offer the facility to control the conditions dealing with our plants irrespective of where you are located, as everything happens over the cloud. Nonetheless, to provide a better overview of our data, we have analyzed our gathered data to learn insights and check whether the optimal indoor conditions match our plants’ needs. Compared to similar works, our study focused on building an affordable IoT platform for smart agriculture that can be specially used for the indoor environment; highly useable for urban farming to save cost and time considering our platform’s affordability and real-time management facilities. We have tested and applied our platform for remote monitoring of plants, soil, and environmental conditions, and based on results from simulations and analyses of previously stored data; our platform could be used to generate important analytics of real-time monitoring, enabling choices and actions such as managing the irrigation system or creating alters, for example. Throughout our experiment, only a few limitations we have noted, such as keeping a constant power supply and keeping the wireless connection stable, as there is no way to communicate with the cloud if one such factor is missing.

Overall, our results indicated that our plants are growing well, and the system is reliable; however, more tests are required to further determine the validity of our system before deploying elsewhere, as site-specific calibration is needed for sensors before deploying. In addition, our prototype system can be further extended with more functionalities and can be upgraded and customized according to the context, such as monitoring and controlling conditions of soil-based and soil-less farming. Examples of this would be the requirement to provide adequate ventilation with automated ventilation fans that work based on the environmental conditions and monitor the visual feed of plants through our platform, which could be developed to a greater extent with real-time decision-making capabilities. For our experiment, we have used Arduino UNO and NodeMCU microcontrollers in conjunction, but our current system can be further upgraded and can connect with more sensors with low-cost microcontrollers like Arduino Mega, which has 16 analog signals as opposed to Arduino UNO. With the comparison of similar work, we elaborated that our work uses more sensors than any other work owing to the integration of two interconnected microcontrollers, which would be ideal for monitoring all sorts of conditions pertaining to the plantation. On the other hand, we also have the option of using the Raspberry Pi Model, which is compatible with the system that is already in place, as it delivers many features in terms of the speed of the CPU, memory, and networking, where it can also act as an edge gateway. Doing so would allow us to perform real-time analytics at the network edge with AI and subsequently execute actions on its own based on the analytics, such as by monitoring the real-time feed, identifying any pests or diseases, and alerting farmers, as we explained in the future work section. In summary, in our research, we have implemented an affordable crop management platform for managing crop conditions in indoor environments. We believe this would pave the way for the design of more smart agricultural solutions, especially for urban farming, to save precious time and cost.

## Data availability statement

The raw data supporting the conclusions of this article will be made available by the authors, without undue reservation.

## Author contributions

NT - Writing and experiment. Others – Supervision. All authors contributed to the article and approved the submitted version.

## References

[B1] Abbas F. N. Saadoon M. I. M. Abdalrdha Z. K. Abud E. N. (2020). Capable of gas sensor MQ-135 to monitor the air quality with arduino uno. Int. J. Eng Res. Technol. 13 (10), 2955–2959. doi: 10.37624/IJERT/13.10.2020.2955-2959

[B2] Ahmed M. A. Gallardo J. L. Zuniga M. D. Pedraza M. A. Carvajal G. Jara N.. (2022). LoRa based IoT platform for remote monitoring of Large-scale agriculture farms in Chile. Sensors 22 (8), 2824. doi: 10.3390/s22082824 35458808 PMC9028925

[B3] Akhter R. Sofi S. A. (2022). Precision agriculture using IoT data analytics and machine learning. J. King Saud University-Computer Inf. Sci 34, 5602–5618. doi: 10.1016/j.jksuci.2021.05.013

[B4] Aminulloh L. Sesulihatien W. T. Pramadihanto D. (2019). Extraction of Tomato Growth Model using Greenhouse Monitoring System, in: 2019 International Electronics Symposium (IES). in Presented at the 2019 International Electronics Symposium (IES). (Surabaya, Indonesia: IEEE), 370–375. doi: 10.1109/ELECSYM.2019.8901534

[B5] Arduino S. A. (2015), 372. Arduino. Arduino LLC.

[B6] Ayaz M. Ammad-Uddin M. Sharif Z. Mansour A. Aggoune E. H. M. (2019). Internet-of-Things (IoT)-based smart agriculture: Toward making the fields talk. IEEE Access 7, 129551–129583. doi: 10.1109/ACCESS.2019.2932609

[B7] Badamasi Y. A. (2014). The working principle of an Arduino, in: 2014 11th International Conference on Electronics, Computer and Computation (ICECCO). in Presented at the 2014 11th International Conference on Electronics, Computer and Computation (ICECCO). (Abuja, Nigeria: IEEE), 1–4. doi: 10.1109/ICECCO.2014.6997578

[B8] Banerjee N. Mukherjee S. Mitra A. Sanyal A. Mandal S. T. (2017). Arduino based liquid dispensor system using peristaltic pump (BS Project).

[B9] Bates H. Pierce M. Benter A. (2021). Real-time environmental monitoring for aquaculture using a LoRaWAN-based IoT sensor network. Sensors 21 (23), 7963. 3. doi: 10.3390/s21237963 34883973 PMC8659442

[B10] Carbon Dioxide (CO2): Environmental Health in Minnesota (2022). Available at: https://www.health.state.mn.us/communities/environment/air/toxins/co2.html.

[B11] Charumathi S. Kaviya R.M. Kumariyarasi J. Manisha R. Dhivya P. (2017). Optimization and control of hydroponics agriculture using IOT. Asian Journal of Applied Science and Technology (AJAST) 1 (2), 96–98.

[B12] Chlingaryan A. Sukkarieh S. Whelan B. (2018). Machine learning approaches for crop yield prediction and nitrogen status estimation in precision agriculture: A review. Comput. Electron. Agric. 151, 61–69. doi: 10.1016/j.compag.2018.05.012

[B13] Cordeiro M. Markert C. Araújo S. S. Campos N. G. Gondim R. S. da Silva T. L. C.. (2022). Towards smart farming: Fog-enabled intelligent irrigation system using deep neural networks. Future Generation Comput. Syst. 129, 115–124. doi: 10.1016/j.future.2021.11.013

[B14] Cucus A. Febrianti M. S. (2017). Implementation of Sensor Ph Meter, Ec Meter and Temperature on Smart Vertical Agriculture System.

[B15] Doshi J. Patel T. kumar Bharti S. (2019). Smart farming using IoT, a solution for optimally monitoring farming conditions. Proc. Comput. Sci. 160, 746–751. doi: 10.1016/j.procs.2019.11.016

[B16] Eller H. Denoth A. (1996). A capacitive soil moisture sensor. J. Hydrology 185 (1-4), 137–146. doi: 10.1016/0022-1694(95)03003-4

[B17] Forcén-Muñoz M. Pavón-Pulido N. López-Riquelme J. A. Temnani-Rajjaf A. Berríos P. Morais R.. (2021). Irriman platform: Enhancing farming sustainability through cloud computing techniques for irrigation management. Sensors 22 (1), 228. doi: 10.3390/s22010228 35009770 PMC8749527

[B18] Gay W. (2018). “DHT11 sensor,” in Advanced raspberry pi(Apress, Berkeley, CA: Springer), 399–418. doi: 10.1007/978-1-4842-3948-3_22

[B19] GlobeNewswire (2022). Available at: https://www.globenewswire.com/news-release/2021/10/18/2315821/0/en/Smart-Agriculture-Market-Size-Globally-Estimated-to-Reach-USD-22-5-Bn-with-8-9-CAGR-by-2026-Facts-Factors.html.

[B20] Growing tomatoes from seed: How, when and ideal temperatures (2022). Available at: https://dengarden.com/gardening/planting-tomato-seeds.

[B21] Hasanaj R. Abuhemidan A. (2019). Air-quality sensor with 10-years lifespan. Available at:https://www.diva-portal.org/smash/get/diva2:1355102/FULLTEXT01.pdf.

[B22] Herman H. Adidrana D. Surantha N. Suharjito S. (2019). Hydroponic nutrient control system based on Internet of things. CommIT (Communication Inf. Technology) J. 13 (2), 105–111. doi: 10.21512/commit.v13i2.6016

[B23] How to grow tomatoes indoors (2022) The spruce. Available at: https://www.thespruce.com/growing-organic-tomatoes-indoors-2539817.

[B24] Junior F. M. R. Bianchi R. A. Prati R. C. Kolehmainen K. Soininen J. P. Kamienski C. A. (2022). Data reduction based on machine learning algorithms for fog computing in IoT smart agriculture. Biosyst. Eng 223, 142–158. doi: 10.1016/j.biosystemseng.2021.12.021

[B25] Kagita M. K. Thilakarathne N. Bojja G. R. Kaosar M. (2021). A lossless compression technique for Huffman-based differential encoding in IoT for smart agriculture. Int. J. Uncertainty Fuzziness Knowledge-Based Syst. 29 (Supp. 02), 317–332. doi: 10.1142/S0218488521400171

[B26] Kashyap M. Sharma V. Gupta N. (2018). Taking MQTT and NodeMcu to IOT: communication in Internet of things. Proc. Comput. Sci. 132, 1611–1618. doi: 10.1016/j.procs.2018.05.126

[B27] Khattab A. Abdelgawad A. Yelmarthi K. (2016). “Design and implementation of a cloud-based IoT scheme for precision agriculture,” in 2016 28th International Conference on Microelectronics (ICM). Presented at the 2016 28th International Conference on Microelectronics (ICM). (Giza, Egypt: IEEE), 201–204. doi: 10.1109/ICM.2016.7847850

[B28] Kour K. Gupta D. Gupta K. Dhiman G. Juneja S. Viriyasitavat W.. (2022). Smart-Hydroponic-Based framework for saffron cultivation: A precision smart agriculture perspective. Sustainability 14 (3), 1120. doi: 10.3390/su14031120

[B29] Latha N. A. Murthy B. R. Kumar K. B. (2016). Distance sensing with ultrasonic sensor and arduino. Int. J. Advance Research Ideas Innov. Technol. 2 (5), 1–5.

[B30] Lee J. Kim S. Lee S. Choi H. Jung J. (2014). A study on the necessity and construction plan of the internet of things platform for smart agriculture. J. Korea multimedia Soc. 17 (11), 1313–1324. doi: 10.9717/kmms.2014.17.11.1313

[B31] Leyva R. Constán-Aguilar C. Sánchez-Rodríguez E. Romero-Gámez M. Soriano T. (2015). Cooling systems in screenhouses: Effect on microclimate, productivity and plant response in a tomato crop. Biosyst. Eng. 129, 100–111. doi: 10.1016/j.biosystemseng.2014.09.018

[B32] Luis Bustamante A. Patricio M. A. Molina J. M. (2019). Thinger. io: An open-source platform for deploying data fusion applications in IoT environments. Sensors 19 (5), 1044. doi: 10.3390/s19051044 30823643 PMC6427624

[B33] Marcu I. Drăgulinescu A. M. Florea C. Bălăceanu C. Dobrea M. A. Suciu G. (2020). Agricultural data fusion for smartagro telemetry system. Adv. sci. technol. eng. syst J. 5, 1266–1272. doi: 10.25046/aj0505152

[B34] Max J. F. Horst W. J. Mutwiwa U. N. Tantau H. J. (2009). Effects of greenhouse cooling method on growth, fruit yield and quality of tomato *(Solanum lycopersicum* l.) in a tropical climate. Scientia Hortic. 122 (2), 179–186. doi: 10.1016/j.scienta.2009.05.007

[B35] Mehra M. Saxena S. Sankaranarayanan S. Tom R. J. Veeramanikandan M. (2018). IoT based hydroponics system using deep neural networks. Comput. Electron. Agric. 155, 473–486. doi: 10.1016/j.compag.2018.10.015

[B36] Mekala M. S. Viswanathan P. (2017). A Survey: Smart agriculture IoT with cloud computing, in: 2017 International Conference on Microelectronic Devices, Circuits and Systems (ICMDCS). in Presented at the 2017 International conference on Microelectronic Devices, Circuits and Systems (ICMDCS). (Vellore: IEEE), 1–7. doi: 10.1109/ICMDCS.2017.8211551

[B37] Mohammed M. Riad K. Alqahtani N. (2021). Efficient iot-based control for a smart subsurface irrigation system to enhance irrigation management of date palm. Sensors 21 (12), 3942. doi: 10.3390/s21123942 34201041 PMC8228936

[B38] Mohanraj I. Ashokumar K. Naren J. (2016). Field monitoring and automation using IOT in agriculture domain. Proc. Comput. Sci. 93, 931–939. doi: 10.1016/j.procs.2016.07.275

[B39] Montazeaud G. Langrume C. Moinard S. Goby C. Ducanchez A. Tisseyre B.. (2021). Development of a low cost open-source ultrasonic device for plant height measurements. Smart Agric. Technol. 1, 100022. doi: 10.1016/j.atech.2021.100022

[B40] O’Grady M. J. Langton D. O’Hare G. M. P. (2019). Edge computing: A tractable model for smart agriculture? Artif. Intell. Agric. 3, 42–51. doi: 10.1016/j.aiia.2019.12.001

[B41] Parihar Y. S. (2019). Internet Of things and nodemcu. J. Emerging Technol. Innovative Research 6(6) p.1085, 19. Available at: https://www.researchgate.net/profile/Yogendra-Singh-Parihar/publication/337656615_Internet_of_Things_and_Nodemcu_A_review_of_use_of_Nodemcu_ESP8266_in_IoT_products/links/5e29767b4585150ee77b868a/Internet-of-Things-and-Nodemcu-A-review-of-use-of-Nodemcu-ESP8266-in-IoT-products.pdf

[B42] Patil N. Patil S. Uttekar A. Suryawanshi A.R. (2020). Monitoring of hydroponics system using IoT technology. International Research Journal of Engineering and Technology (IRJET) 07 (06), 5.

[B43] Quy V. K. Hau N. V. Anh D. V. Quy N. M. Ban N. T. Lanza S.. (2022). IoT-enabled smart agriculture: Architecture, applications, and challenges. Appl. Sci. 12 (7), 3396. doi: 10.3390/app12073396

[B44] Ratnaparkhi S. Khan S. Arya C. Khapre S. Singh P. Diwakar M.. (2020). Smart agriculture sensors in IOT: A review. Materials Today: Proc, S2214785320387447. doi: 10.1016/j.matpr.2020.11.138

[B45] Reddy K. S. P. Roopa Y. M. LN K. R. Nandan N. S. (2020). IoT based Smart Agriculture using Machine Learning, in: 2020 Second International Conference on Inventive Research in Computing Applications (ICIRCA). in Presented at the 2020 Second International Conference on Inventive Research in Computing Applications (ICIRCA). (Coimbatore, India: IEEE), 130–134 doi: 10.1109/ICIRCA48905.2020.9183373q.

[B46] Saha R. Biswas S. Sarmah S. Karmakar S. Das P. (2021). A working prototype using DS18B20 temperature sensor and arduino for health monitoring. SN Comput. Sci. 2 (1), 1–21. doi: 10.1007/s42979-020-00434-2 PMC7802410 33458699

[B47] Salim G. M. Ismail H. Debnath N. Nadya A. (2015). Optimal light power consumption using LDR sensor, in: 2015 IEEE International Symposium on Robotics and Intelligent Sensors (IRIS). in Presented at the 2015 IEEE International Symposium on Robotics and Intelligent Sensors (IRIS). (Langkawi, Malaysia: IEEE), 144–148. doi: 10.1109/IRIS.2015.7451601

[B48] Schwarz D. Thompson A. J. Kläring H. P. (2014). Guidelines to use tomato in experiments with a controlled environment. Front. Plant Sci. 5, 625. doi: 10.3389/fpls.2014.00625 25477888 PMC4235429

[B49] Shamshiri R. R. Jones J. W. Thorp K. R. Ahmad D. Man H. C. Taheri S. (2018). Review of optimum temperature, humidity, and vapour pressure deficit for microclimate evaluation and control in greenhouse cultivation of tomato: a review. Int. agrophysics 32 (2), 287–302. doi: 10.1515/intag-2017-0005

[B50] Sihombing P. Peranginangin B. Sitompul D. Rivaldo R. (2019). Tools for detecting and control of soil pH by probe sensor based on android. J. Phys. 1230 (1), 012033. doi: 10.1088/1742-6596/1230/1/012033

[B51] Smart agriculture (2022). Available at: https://www.statista.com/topics/4134/smart-agriculture/.

[B52] Stafford T. M. (2015). Indoor air quality and academic performance. J. Environ. Economics Manage. 70, 34–50. doi: 10.1016/j.jeem.2014.11.002

[B53] Suanpang P. Jamjuntr P. (2019). A smart farm prototype with an Internet of things (IoT) case study: Thailand. technology 5 (12), 15. doi: 10.18178/joaat.6.4.241-245

[B54] Suciu G. Istrate C. I. Diţu M. C. (2019). Secure smart agriculture monitoring technique through isolation, in: 2019 Global IoT Summit (GIoTS). Presented at the 2019 Global IoT Summit (GIoTS) (Aarhus, Denmark: IEEE), 1–5. doi: 10.1109/GIOTS.2019.8766433

[B55] Sunday A. O. Constance E. E. Abdullahi A. B. U. H. Ehiremen I. A. Ebehiremen O. P. (2020). A dual channel home automation and security system using finite state machine. J. Comput. Sci. Control Syst. 13 (2).

[B56] Suryawinata H. Purwanti D. Sunardiyo S. (2017). Sistem monitoring pada panel surya menggunakan data logger berbasis ATMega 328 dan real time clock DS1307. Jurnal teknik elektro 9 (1), 30–36.

[B57] Sushanth G. Sujatha S. (2018). IOT Based Smart Agriculture System, in: 2018 International Conference on Wireless Communications, Signal Processing and Networking (WiSPNET) in Presented at the 2018 International Conference on Wireless Communications, Signal Processing and Networking (WiSPNET). (Chennai: IEEE), 1–4. doi: 10.1109/WiSPNET.2018.8538702

[B58] The digitization of the European Agricultural Sector (2022) Shaping europe’s digital future. Available at: https://digital-strategy.ec.europa.eu/en/policies/digitisation-agriculture.

[B59] Theparod T. Harnsoongnoen S. (2022). Narrow-band light-emitting diodes (LEDs) effects on sunflower *(Helianthus annuus)* sprouts with remote monitoring and recording by Internet of things device. Sensors 22 (4), 1503. doi: 10.3390/s22041503 35214417 PMC8877001

[B60] Thilakarathne N. N. Bakar M. S. A. Abas P. E. Yassin H. (2022). A cloud enabled crop recommendation platform for machine learning-driven precision farming. Sensors 22 (16), 6299. doi: 10.3390/s22166299 36016060 PMC9412477

[B61] Thilakarathne N. N. Yassin H. Bakar M. S. A. Abas P. E. (2021). Internet of Things in Smart Agriculture: Challenges, Opportunities and Future Directions in 2021 IEEE Asia-Pacific Conference on Computer Science and Data Engineering (CSDE). Presented at the 2021 IEEE Asia-Pacific Conference on Computer Science and Data Engineering (CSDE), (Brisbane, Australia: IEEE), 1–9. doi: 10.1109/CSDE53843.2021.9718402

[B62] Thinger.io (2022). Available at: https://thinger.io/.

[B63] Trilles S. Torres-Sospedra J. Belmonte Ó. Zarazaga-Soria F. J. González-Pérez A. Huerta J. (2020). Development of an open sensorized platform in a smart agriculture context: A vineyard support system for monitoring mildew disease. Sustain. Computing: Inf. Syst. 28, 100309. doi: 10.1016/j.suscom.2019.01.011

[B64] Walter A. Finger R. Huber R. Buchmann N. (2017). Smart farming is key to developing sustainable agriculture. Proc. Natl. Acad. Sci. 114 (24), 6148–6150. doi: 10.1073/pnas.1707462114 28611194 PMC5474773

